# Oxidation-resistant all-perovskite tandem solar cells in substrate configuration

**DOI:** 10.1038/s41467-023-37492-y

**Published:** 2023-03-31

**Authors:** Yurui Wang, Renxing Lin, Xiaoyu Wang, Chenshuaiyu Liu, Yameen Ahmed, Zilong Huang, Zhibin Zhang, Hongjiang Li, Mei Zhang, Yuan Gao, Haowen Luo, Pu Wu, Han Gao, Xuntian Zheng, Manya Li, Zhou Liu, Wenchi Kong, Ludong Li, Kaihui Liu, Makhsud I. Saidaminov, Lijun Zhang, Hairen Tan

**Affiliations:** 1 National Laboratory of Solid State Microstructures, College of Engineering and Applied Sciences, Frontiers Science Center for Critical Earth Material Cycling, Nanjing University, Nanjing, 210023 China; 2 State Key Laboratory of Superhard Materials, Key Laboratory of Automobile Materials of MOE, College of Materials Science and Engineering, Jilin University, Changchun, China; 3 Department of Chemistry, University of Victoria, Victoria, BC Canada; 4 State Key Laboratory for Mesoscopic Physics, Frontiers Science Center for Nano-optoelectronics, School of Physics, Peking University, Beijing, China

**Keywords:** Solar energy and photovoltaic technology, Optical materials

## Abstract

The commonly-used superstrate configuration (depositing front subcell first and then depositing back subcell) in all-perovskite tandem solar cells is disadvantageous for long-term stability due to oxidizable narrow-bandgap perovskite assembled last and easily exposable to air. Here we reverse the processing order and demonstrate all-perovskite tandems in a substrate configuration (depositing back subcell first and then depositing front subcell) to bury oxidizable narrow-bandgap perovskite deep in the device stack. By using guanidinium tetrafluoroborate additive in wide-bandgap perovskite subcell, we achieve an efficiency of 25.3% for the substrate-configured all-perovskite tandem cells. The unencapsulated devices exhibit no performance degradation after storage in dry air for 1000 hours. The substrate configuration also widens the choice of flexible substrates: we achieve 24.1% and 20.3% efficient flexible all-perovskite tandem solar cells on copper-coated polyethylene naphthalene and copper metal foil, respectively. Substrate configuration offers a promising route to unleash the commercial potential of all-perovskite tandem solar cells.

## Abstract (web summary)

The superstate configuration in all-perovskite tandem solar cells is disadvantageous for long-term stability. Here, the authors reverse the processing order and demonstrate substrate configuration to bury oxidizable narrow-bandgap perovskites, and achieve efficiency of 25.3% with long stability.

## Introduction

Monolithic all-perovskite tandem solar cells include a front subcell with ~1.8 eV wide-bandgap (WBG) perovskite and a back subcell with ~1.2 eV narrow-bandgap (NBG) perovskite^1–3^. All-perovskite tandem solar cells have achieved a record certified efficiency of 26.4%, exceeding the single-junction perovskite solar cells (PSCs)^1,2,4,5^. But oxygen-related instability remains a key challenge for their commercialization^6–8^. Encapsulation can prevent oxygen-induced degradation in the active layers or electrodes; however, oxidation can still occur during the module processing and/or air leakage during operation. For flexible all-perovskite tandem solar cells, polymer substrates with high transparency are typically needed, limiting the choice of substrates and increasing the cost of flexible devices^9^. Therefore, new device structures that can allow higher resistance to oxygen and lower material cost for flexible devices are needed.

**Fig. 1:**
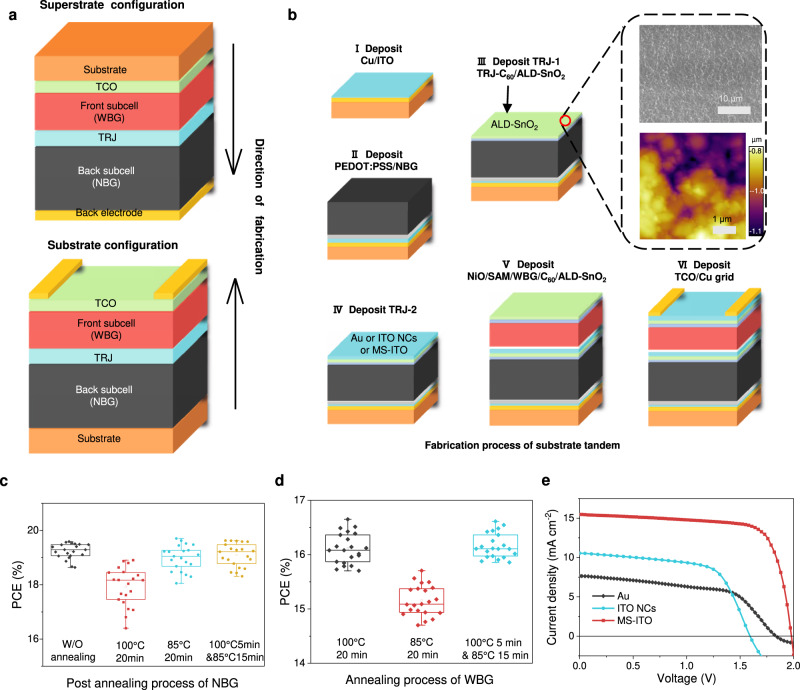
Design and fabrication process of all-perovskite tandem solar cells in substrate configuration. **a** Device structure of superstrate- and substrate-configured all-perovskite tandem solar cells. **b** Fabrication process of all-perovskite tandem solar cells in substrate configuration. **c** Effect of post-annealing conditions on semitransparent narrow-bandgap PSCs. **d** Effect of annealing conditions on semitransparent wide-bandgap PSCs. There are 21 devices for each type in b and c, and the box lines indicate the standard deviation, and the centre represents the mean value. **e** *J–V* curves of substrate-configurated all-perovskite tandem solar cells with Au or ITO nanocrystals (ITO NCs) or magnetron-sputtered ITO (MS-ITO) as the recombination layer.

Reported all-perovskite tandems^2,3,10–13^, either rigid or flexible, typically use a “superstrate configuration” structure (Fig. 1a)^14,15^. In this structure, WBG front subcell is deposited first on a rigid or flexible transparent conductive substrate (e.g., glass/ITO or PEN/ITO), followed by the tunneling recombination junction (TRJ), NBG back subcell, and metal back electrode. In this configuration, sunlight first passes through the front transparent substrate, and then is absorbed in the front subcell and the back subcell in sequence. This structure has been widely implemented and is compatible with low-cost solution processing.

However, externally exposed NBG perovskites, made of mixed lead-tin (Pb-Sn), are more sensitive to oxygen than WBG perovskites buried at the bottom, making the superstrate configuration an inherent disadvantage to device stability. Pb-based PSCs have been widely proven to be stable in air for a period of time without degradation of performance^16,17^. But this is not the case for Pb-Sn-based perovskites: Sn^2+^ in NBG perovskite is oxidized to form Sn^4+^ on a minute scale in the presence of oxygen, leading to high trap densities and short diffusion lengths, and thus device degradation^18,19^. The oxidation of Sn^2+^ can be inhibited by comproportionation^2^ or reducing additives like tin (II) fluoride (SnF_2_)^20–22^ or others^23,24^. This strategy improves the quality of the precursor solution and device performance. Coating a thin layer of metal oxide (e.g., SnO_2_) made by atomic layer deposition (ALD) on top of Pb-Sn perovskite layer can by some extent prevent the penetration of oxygen into the absorber layer and thus improve the air stability in tandem devices, but this only provides limited protection^24,25^. The oxidation of Sn^2+^ remains a major challenge for the long-term stability of all-perovskite tandem solar cells.

Here we sought to devise all-perovskite tandem solar cells with a substrate-configured device structure (illustrated in Fig. 1a), in which easily oxidizable NBG back subcell is deposited first and is buried at the bottom of the tandem device. In the substrate configuration, TRJ and front subcell offer a self-encapsulation for the NBG perovskite, effectively preventing the penetration of oxygen into the mixed Pb-Sn perovskite absorber layer. In addition, this configuration does not demand the substrate to be transparent, thus widening the choice from transparent substrates (e.g., glass, polyethylene terephthalate (PET)^26^, polyethylene naphthalate (PEN)^27^ and polyimide (PI)^28^) to opaque and high-temperature tolerant flexible polymers, stainless steel^29^ and metal foils^30^. This allows lower cost for lightweight and flexible all-perovskite tandem solar modules^13^. By adopting a substrate configuration, we demonstrated rigid all-perovskite tandem solar cells with a PCE of 25.3%. The unencapsulated devices exhibited impressive tolerance to oxygen and maintained their initial performance when stored in dry air for more than 1000 hours. The encapsulated devices maintained 100% of their initial performance after 600 hours of operation at maximum power point in ambient conditions. The substrate configuration also enabled the fabrication of efficient flexible all-perovskite tandem solar cells on metal-coated polymer substrates and metal foils.

## Results

### Fabrication of substrate-configured tandems

The fabrication process of substrate-configured all-perovskite tandem solar cell is illustrated in Fig. 1b. We chose Cu/ITO as the back electrode; this also eased the transfer of devices to metal substrates, as we will show below. In step I, a thin layer of copper (Cu, 80 nm) was deposited on glass by thermal deposition. A layer of ITO (15 nm) was then sputtered on the substrate to prevent any potential reactions between Cu and perovskite. In step II, the back subcell in PEDOT: PSS/NBG perovskite/C_60_ structure was deposited using our previously reported process^2,24^. In step III, SnO_2_ layer was deposited by ALD (ALD-SnO_2_) to act as part of TRJ and as a solvent barrier layer. Scanning electron microscopy (SEM) and atomic force microscopy (AFM) were used to observe the surface of ALD-SnO_2_ layer deposited on top of the back subcell (Fig. 1b inset). Supplementary Fig. 1 shows the SEM and AFM images of ALD-SnO_2_ film on Glass/ITO/NiO/WBG perovskite/C_60_, the structure of WBG subcell used in superstrate-configured all-perovskite tandems^2,24^. Both configurations showed compact and pinhole-free ALD-SnO_2_ films, but significantly higher root-mean-square (RMS) roughness on NBG perovskites (87.9 nm) as compared to that on WBG perovskites (17.2 nm). This is because the thick NBG perovskites tend to have larger grains, resulting in a rougher surface; this makes the construction of TRJ challenging^31^.

In TRJ, an additional recombination layer (RL) is required to enable efficient charge recombination^32^. In step IV, we deposited three commonly-used RLs on ALD-SnO_2_: a thin layer of gold (Au) by thermal evaporation^2^, spin-coated ITO nanocrystals (ITO NCs)^25^ and magnetron-sputtered ITO (MS-ITO)^10^. All three RLs allowed a good Ohmic contact when the TRJ was deposited on smooth glass substrates, although with a slight difference in the resistance (Supplementary Fig. 2). However, the substrate-configured tandem devices with Au or ITO NCs as the RL showed “S-shaped” *J-V* curves and hence low *FF* values **(**Fig. 1e**)**. This indicates a Schottky contact formed at TRJ, resulting in poor electron-hole recombination^2^. We speculate that neither Au nor ITO NCs can spread evenly on the rough surface of NBG perovskite/C_60_/ALD-SnO_2_, although they can cover the WBG perovskite/C_60_/ALD-SnO_2_ smooth surface well (Supplementary Fig. 3). We thus chose MS-ITO for the RL: an added benefit for this choice is that inorganic oxides offer longer-term stability than metals used in TRJ^25^. The dense ALD-SnO_2_ layer prevents the damage caused by sputtering, and using a thin layer MS-ITO down to 20 nm reduced the parasitic absorption (Supplementary Fig. 4).

In step V, the front subcell with a structure of NiO/SAM/WBG perovskite/C_60_/ALD-SnO_2_ was deposited, in which NiO is nickel oxide and SAM is a self-assembled molecule. Solvent penetration and thermal damages are two major issues while building front subcells on the back cells. As shown in Fig. 1c and Supplementary Fig. 5, the PCE of the NBG PSCs decreased to 85% of its initial value after 20 min of post-annealing at 100 °C. The decreased PV performance is mainly ascribed to the volatile nature of methylammonium (MA)^31,33^ and an unfavorable thermal-induced reaction at the PEDOT: PSS/perovskite interface^34,35^. Efforts have been devoted to developing MA-free NBG perovskites and thermally stable HTL materials to improve the thermal stability of NBG PSCs^25,36–38^. We found that relatively low post annealing temperature, i.e. 85 °C, minimizes damage to the NBG-PSCs, but this decreased the PCE of WBG cells (Fig. 1d) likely due to hindered crystallization^39^. Therefore, we combined a lower temperature (85 °C, 15 min) annealing with a higher temperature but shorter time annealing processes (100 °C, 5 min) for the WBG perovskite^40,41^. By using this strategy, we were able to balance the performance of WBG and NBG subcells simultaneously.

Finally, in step VI, we deposited indium zinc oxide (IZO) by magnetron sputtering on top of the device as the transparent electrode. The tandem cells with MS-ITO as the RJ exhibited a PCE of 22.7%, with a *V*_*oc*_ of 1.980 V, a *J*_*sc*_ of 15.4 mA cm^−2^, and a low *FF* of 74.1%. The *V*_*oc*_ of tandem devices is the sum of *V*_*oc*_ values of the two subcells. The *FF* of a current-matched tandem device does correlate with the *FF* of each subcell^42^. Supplementary Fig. 6 shows that semitransparent NBG subcells (glass/Cu/ITO/PEDOT: PSS/NBG-perovskite/C_60_/SnO_2_/ITO/Cu-grid) can reach a PCE of 19.6% with a high *FF* of 78.7%. We, therefore, anticipate that further improvement of substrate-configured tandem cells should improve the *V*_*oc*_ and *FF* in the semitransparent WBG subcells.

### Semitransparent WBG subcells with GuaBF_4_ additive

**Fig. 2:**
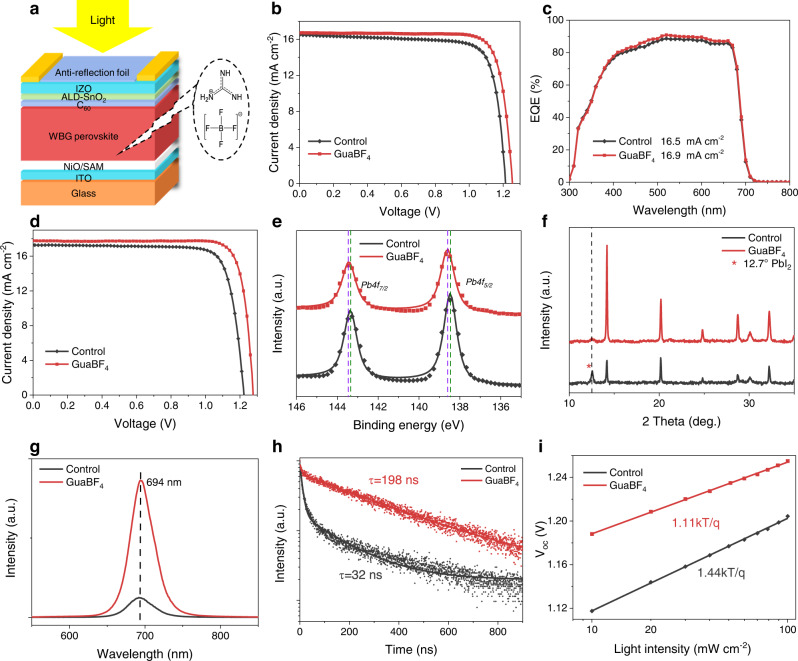
Characterization of WBG perovskite films and semitransparent solar cells with GuaBF_4_ additive. **a** Device structure of semitransparent WBG-PSCs. **b** *J–V* curves of champion control and GuaBF_4_ semitransparent WBG-PSCs. **c** EQE spectra of control and GuaBF_4_ semitransparent WBG-PSCs. **d** *J–V* curves of champion control and GuaBF_4_ WBG-PSCs in superstrate configuration (device structure shown in Supplementary Fig. 7a). **e** The *Pb 4f*_*5/2*_ and *Pb 4f*_*7/2*_ XPS spectra of control and GuaBF_4_ perovskite films. **f** The XRD pattern of control and GuaBF_4_ perovskite films. **g** Steady-state PL spectra of control and GuaBF_4_ perovskite films. **h** TRPL spectra of perovskite films deposited on Glass/ITO. **i** *V*_*oc*_ as a function of light intensity plots of the semitransparent WBG-PSCs.

We fabricated semitransparent WBG PSCs with a perovskite composition of FA_0.8_Cs_0.2_Pb(I_0.6_Br_0.4_)_3_ (~1.77 eV) and NiO/SAM as the HTL^43^ (Fig. 2a). However, such semitransparent devices showed low performance mainly due to the low *FF* and *V*_*oc*_ values, compared to the opaque p-i-n PSCs in superstrate configuration (Table 1). We hence sought to use guanidine tetrafluoroborate (GuaBF_4_) into the perovskite precursor solution. The deep-level defects caused by the high ratio of Br incorporated in WBG perovskites profoundly limit the performance of solar cells^44^. Various large organic cations have been introduced into perovskites for the passivation of halogen defects^45^. Pseudo-halogens have also been reported to inhibit halogen migration^46^. Combined with DFT studies (Supplementary note 1), we reveal that guanidinium (Gua^+^) avoids the unfavorable lattice distortion and is more effective than phenethylammonium (PEA^+^)^47^ and phenylammonium (PA^+^)^5^ in suppressing halogen vacancy defects (Supplementary Figs. 7–8) due to the stronger hydrogen bonding. The Pb-F bond introduced by tetrafluoroborate (BF_4_^-^) leads to an effective passivation for halogen vacancies^48^ (Supplementary Fig. 9). Overall, guanidine tetrafluoroborate (GuaBF_4_) is expected to inhibit the formation of halogen vacancy defects with minimal effect on the perovskite lattice^49^. The optimized concentration of GuaBF_4_ added in perovskite precursor solution was found to be 1.5% molar ratio relative to Pb^2+^. The devices with GuaBF_4_ additive exhibited an obvious improvement in average *V*_*oc*_ and *FF* compared to the control ones (1.20 V versus 1.24 V; 77.9% versus 80.8%, Table 1 and Supplementary Fig. 10), while the average *J*_*sc*_ remained the same. As a result, the GuaBF_4_ devices showed a considerably higher average PCE (16.7%) than the control devices (15.3%). To reduce the primary optical reflection, we attached a commercially available anti-reflection foil^50^ on the top of the substrate-configurated devices (Fig. 2a). The champion GuaBF_4_ device showed a PCE of 17.3% (with *V*_*oc*_ = 1.265 V, *J*_*sc*_ = 16.7 mA cm^−2^ and *FF* = 81.9%), which is higher than that of control device (PCE of 15.8% with *V*_*oc*_ = 1.214 V, *J*_*sc*_ = 16.5 mA cm^−2^ and *FF* = 78.1%) (Fig. 2b). The *J*_*sc*_ value (16.9 mA cm^−2^) extracted by integrating the external quantum efficiency (EQE) curve is in good agreement with the *J–V* characterization (Fig. 2c).

**Table. 1:** PV performance of single-junction WBG PSCs in substrate and superstrate configurations measured under AM1.5 illumination

Device configuration			*V*_*oc*_(V)	*J*_*sc*_(mA cm^−2^)	*FF*(%)	PCE(%)
Substrate (semitransparent, light from IZO)	Average	Control	1.199	16.3	77.9	15.3
		GuaBF_4_	1.244	16.5	80.8	16.7
	Champion	Control	1.214	16.5	78.1	15.8
		GuaBF_4_	1.265	16.7	81.9	17.3
Superstrate (opaque, light from glass)	Average	Control	1.217	17.1	79.5	16.6
		GuaBF_4_	1.263	17.4	82.3	18.4
	Champion	Control	1.221	17.2	81.0	17.2
		GuaBF_4_	1.274	17.7	84.5	19.1

We noted that GuaBF_4_ also improved the performance of WBG PSCs in superstrate configuration (Supplementary Fig. 11). The average *V*_*oc*_ increased from 1.217 V to 1.263 V, while the average *FF* increased from 79.5% to 82.3%. The champion GuaBF_4_ device in superstrate configuration exhibited a PCE of 19.1% (*V*_*oc*_ = 1.274 V, *J*_*sc*_ = 17.7 mA cm^−2^ and FF = 84.5%, Fig. 2d), which is substantially higher than control device (PCE of 17.2% with *V*_*oc*_ = 1.221 V, *J*_*sc*_ = 17.2 mA cm^−2^ and *FF* = 81.0%).

We then studied the effect of GuaBF_4_ additive on perovskite films. X-ray photoelectron spectroscopy (XPS) peaks at 288.5 and 687.3 eV corresponding to Gua (*C1s*)^1^ and *F1s*^51^ confirm the presence of GuaBF_4_ in perovskite films (Supplementary Fig. 12). Meanwhile, the increased binding energy of Pb peaks (Fig. 2e) suggest a chemical interaction between GuaBF_4_ and the surface of perovskite grains, since GuaBF_4_ can bind with uncoordinated Pb^2+^ and/or fill the point vacancies^51^. As shown in Supplementary Fig. 13, The addition of Gua^+^ only (GuaI) results in reduced binding energy of Pb, which is in contrast to when BF_4_^-^ is used. The ionic radii of BF_4_^-^ (0.218 nm) and I^-^ (0.220 nm) are nearly the same^52^, but BF_4_^-^ weakly hybridizes with the atomic orbitals of Pb^2+^, strengthening covalent bond contribution between BF_4_^-^ and Pb^2+^ ^53^. The increase in binding energy of Pb^2+^ with GuaBF_4_ perovskite film is a concerted effect of Gua^+^ and BF_4_^-^, since they, on their own, have opposite effects on the binding energy of Pb^2+^. This is consistent with our DFT calculations.

SEM images (Supplementary Fig. 14) show no obvious change in the grain size of control (319 nm) and GuaBF_4_ (356 nm) perovskite films. X-ray diffraction (XRD) shows that GuaBF_4_ perovskite film has an enhanced (100) orientation, accompanied by a significantly weakened PbI_2_ diffraction peak at 12.7° peak (Fig. 2f). As indicated by the DFT calculations, GuaBF_4_ inhibits the formation of vacancy and PbI_2_, which helps improve device stability^54^. No obvious shift in XRD peaks was observed, although both Gua^+^ and BF_4_^−^ have shown the potential to be introduced into the perovskite lattices^45,48,55,56^, indicating that GuaBF_4_ mainly exists on the surface and grain boundaries of the perovskite film. Contact angle (CA) tests show that perovskite film with GuaBF_4_ (CA = 75°) has a larger contact angle than control sample without GuaBF_4_ (CA = 48°), which represents a better surface hydrophobicity (Supplementary Fig. 15). Both control and GuaBF_4_ films exhibited photoluminescence (PL) peaks at 694 nm. The latter showed a much stronger PL intensity, indicating reduced non-radiative recombination due to lower trap density in GuaBF_4_ sample^57^ (Fig. 2g). Time-resolved photoluminescence (TRPL) lifetime of the GuaBF_4_ film (198 ns) was longer than that of the control film (32 ns; Fig. 2h) further indicating suppressed carrier trapping upon the introduction of GuaBF_4_. This reduced non-radiative recombination leads to the increased *V*_*oc*_ in the GuaBF_4_ devices^58^. Transient photovoltage (TPV) decay showed that the GuaBF_4_ devices had a longer charge recombination lifetime (τ_rec_) of 15.4 µs compared to the control one with 2.5 µs (Supplementary Fig. 16). The longer τ_rec_ indicates less recombination in the GuaBF_4_ devices^59,60^, consistent with the results obtained from the PL analysis. Light intensity-dependent *V*_*oc*_ analysis shown in Fig. 2i indicates that the GuaBF_4_ device is closer to unity ideality factor than the control device (1.11 for GuaBF_4_ and 1.44 for control). Devices with GuaBF_4_ additive exhibited a lower dark current density than the control devices (Supplementary Fig. 17), consistent with the increase in *V*_*oc*_ and *FF*^20^.

### Performance of substrate-configured all-perovskite tandems

**Fig. 3:**
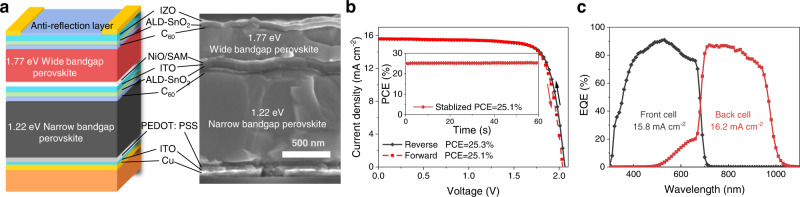
Photovoltaic performance of substrate-configurated all-perovskite tandem solar cells. **a** Device structure and corresponding cross-sectional SEM image of substrate-configurated tandem solar cells. **b**, **c** *J–V* curves, EQE spectra, and MPP tracking (inset **b**) of champion GuaBF_4_ substrate-configurated tandem solar cells.

Motivated by improved *V*_*oc*_ and *FF* in semitransparent WBG PSCs using GuaBF_4_ additive, we fabricated substrate-configured all-perovskite tandems with a device structure shown in Fig. 3a. The thicknesses of WBG and NBG perovskite layers are ~400 nm and ~1100 nm, respectively. The average PCE of tandem devices increased from 22.6% to 24.1% after adding GuaBF_4_ in WBG perovskites (Supplementary Fig. 18). Fig 3b presents the *J–V* curves of the best-performing all-perovskite tandems with GuaBF_4_, the corresponding PV parameters are summarized in Table 2. The champion GuaBF_4_ tandem cell exhibited a high PCE of 25.3% under reverse scan, with a *V*_*oc*_ of 2.041 V, a *J*_*sc*_ of 15.6 mA cm^−2^, and a *FF* of 78.9%. The device showed a minor hysteresis between reverse and forward scans (25.3% versus 25.1%). The PCE from *J–V* sweeps is consistent with the stabilized PCE of 25.1% measured over 60 seconds (Fig. 3b inset). The integrated *J*_*sc*_ values from EQE spectra (Fig. 3c) for the WBG (15.8 mA cm^−2^) and NBG (16.2 mA cm^−2^) subcells are in good agreement with the *J*_*sc*_ determined from *J–V* measurements.

**Table. 2:** PV performance of champion substrate-configured all-perovskite tandems with different substrates measured under AM1.5 illumination

Substrate	Scan direction	*V*_*oc*_(V)	*J*_*sc*_(mA cm^−2^)	*FF*(%)	PCE(%)
Glass (rigid)	Reverse	2.049	15.6	78.7	25.3
	Forward	2.021	15.6	79.5	25.1
PEN/Cu (flexible)	Reverse	2.017	15.6	76.2	24.1
	Forward	2.012	15.5	76.1	23.8
Cu foil (flexible)	Reverse	1.957	14.1	73.4	20.3
	Forward	1.944	14.6	73.5	20.8

### Stability of substrate-configured all-perovskite tandems

**Fig. 4:**
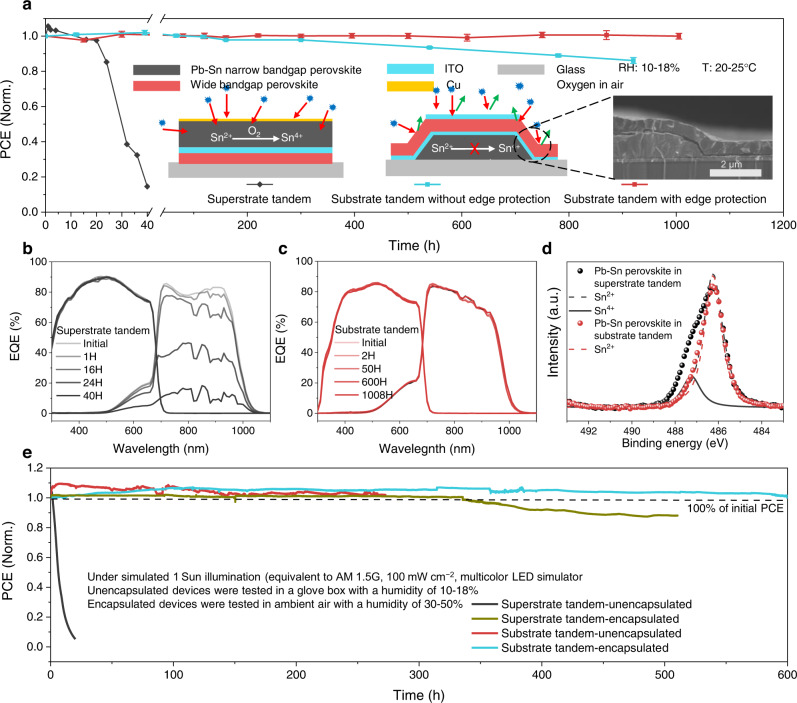
Stability of substrate- and superstrate-configurated all-perovskite tandem solar cells. **a** Dark shelf stability of unencapsulated tandem devices stored in a glovebox filled with dry air (RH < 20%). The inset shows the schematic diagram of the oxidation process of NBG perovskite in superstrate- and substrate-configurated tandems, and a cross-section SEM image of the edge of a substrate-configurated tandem. **b**, **c** EQE spectra of superstrate- and substrate-configurated tandem during the aging process. **d** The *Sn 3d*_*5/2*_ XPS spectra of NBG perovskite layers in superstrate- and substrate-configurated tandems. The superstrate-configurated and substrate-configurated tandems were aged in dry air for 20 and 200 hours, respectively. **e** Operating stability of unencapsulated superstrate- and substrate-configurated tandems, and encapsulated superstrate- and substrate-configurated tandems. The encapsulated device retained 100% of its initial efficiency after 600 hours of operation. All tests were carried out under 1-sun illumination (100 mW cm^–2^) and MPP tracking.

We next compared the stability of superstrate- and substrate-configured tandem solar cells (Fig. 4a). The superstrate-configured tandem devices were fabricated in accordance to architecture shown in Fig. 1a, with MS-ITO as TRJ, and GuaBF_4_ additive in WBG perovskite, and reached the best PCE of 25.6% with a *V*_*oc*_ of 2.034 V, a *J*_*sc*_ of 16.1 mA cm^−2^, and *FF* of 78.2% (Supplementary Fig. 19). For substrate-configured tandems, we used a scraper to remove the edges of NBG back subcell before the deposition of the TRJ and WBG. The edge of the NBG subcell can be “self-encapsulated” by the device itself, which further improves the device stability (Fig. 4a inset). We placed 7 devices for each configuration without encapsulation in a glovebox filled with dry air (relative humidity, RH, below 20%). Unencapsulated superstrate-configured devices showed improved performance in the first 5 hours^2,61,62^, but followed by a severe degradation in the next 40 hours. In contrast, unencapsulated substrate-configurated tandem devices remained stable after being exposure to dry air for more than 250 hours, when using a self-encapsulated process, this value is 1000 hours. Meanwhile, we tracked the EQE of tandem devices to understand the degradation reasons. We found that for superstrate-configurated tandems the EQE of the WBG was stable but the EQE of the NBG subcell decreased dramatically (Fig. 4b). For the substrate-configurated devices, both WBG and NBG subcells did not show noticeable degradation in EQE following exposure to air for more than 1000 hours (Fig. 4c). To investigate the oxidation of NBG perovskites in air, we then exposed superstrate and substrate-configured all-perovskite tandems to dry air for 20 hours and 200 hours, respectively. We carried out XPS characterization of the NBG perovskite layers after removing the top layers by sticky tape and p-dichlorobenzene (Fig. 4d). Two obvious peaks at 486.2 eV and 487.1 eV, corresponding to Sn^2+^ and Sn^4+^, respectively, were observed for superstrate-configurated devices after exposure to air for 20 hours. In contrast, no significant Sn^4+^ signal peak was detected for the substrate-configurated tandems after being exposed to air even for 10× longer period (200 hours).

We also investigated the operating stability of unencapsulated tandems under simulated 1-sun illumination and maximum power point (MPP) operation in a glovebox filled with dry air (Fig. 4e). The PCEs of both superstrate- and substrate-configured tandems increased slightly in the first few hours. The substrate-configurated tandems did not exhibit any noticeable degradation after more than 200 hours of continuous operation. However, the unencapsulated superstrate-configurated tandems degraded dramatically within 20 hours of continuous operation. Furthermore, the encapsulated substrate-configurated tandems retained 100% of their initial performance following 600 hours of continuous operation, while the encapsulated superstrate-configurated tandems experienced severe degradation after 340 hours of operation. We then tested the damp-heat stability (85 °C and relative humidity of 85%) of tandem devices with two different structures. Supplementary Fig. 20 shows that substrate-configured tandem devices exhibited improved stability under temperature of 85 °C and RH of 85% conditions. The substrate-configured tandems also showed better stability under high moisture and light soaking conditions (see Supplementary Note 2 and Supplementary Note 3).

### Flexible substrate-configured tandems on opaque substrates

**Fig. 5:**
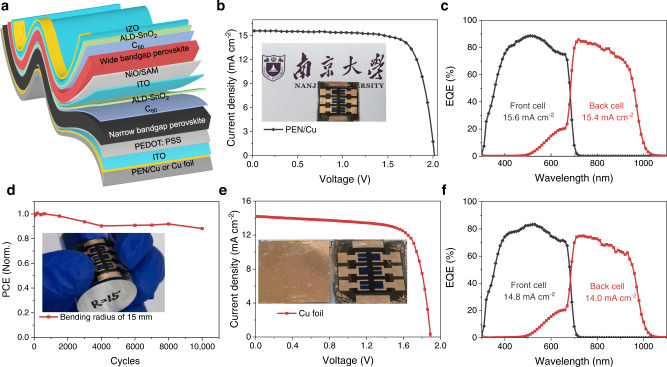
All-perovskite tandem solar cells on flexible opaque substrates. **a** Device structure of a flexible substrate-configurated tandem. The substrate can be PEN or Cu-foils here. **b**, **c** *J–V* curves, and EQE spectra of champion flexible substrate-configurated tandems. The inset shows the image of a flexible substrate-configurated tandem with an active area of 0.09 cm^2^. **d** Bending tests of flexible substrate-configurated tandem cells under a bending radius of 15 mm. The inset shows the digital image of a flexible substrate-configurated tandem under bending. **e**, **f** *J–V* curves, and EQE spectra of a Cu-foils flexible substrate-configurated tandems. The inset shows the image of a flexible substrate-configurated tandem using Cu-foils as substrate.

Due to the change of incident light direction, the substrate configuration widens the choice of flexible substrates, allowing the use of opaque materials such as metal foils and metal-coated polymer foils. We first fabricated flexible tandem solar cells on Cu-coated PEN substrates (device structure shown in Fig. 5a). The champion flexible substrate-configurated tandem had a PCE of 24.1% under reverse scan. The integrated *J*_*sc*_ values of the WBG and NBG subcells from EQE spectra (Fig. 5c) are 15.6 and 15.4 mA cm^−2^, respectively, consistent with the *J*_*sc*_ determined from the *J–V* measurements. We further carried out bending durability tests for flexible tandems. The flexible devices retained over 88% of their initial performance following 10,000 bending cycles with a bending curvature radius of 15 mm (Fig. 5d). When the bending radius was 12 mm, 10 mm and 8 mm, 90%, 78%, and 62% of the initial performance can be retained after 1000 bending cycles, respectively (Supplementary Fig. 21). Thick perovskite absorbers, as required for effective light absorption, used in those flexible tandems could be the main reason for the reduced mechanical stability under smaller bending radius. Using thinner absorbers is expected to significantly improve the flexibility, and nanophotonic strategies can be implemented to compensate for the inherent optical loss caused by the reduction of perovskite thickness. Advanced light-trapping techniques^63–66^ will help reduce the thickness of WBG and NBG (and thus improve flexibility) while maintaining or even improving the efficiency of flexible tandems.

Furthermore, we fabricated tandem cells on Cu foils (thickness 25 μm) as the substrate, achieving a PCE of 20.3% (Fig. 5e). The integrated *J*_*sc*_ values of the WBG and NBG subcells from EQE spectra (Fig. 5f) are 14.8 and 14.0 mA cm^−2^, respectively. The perovskite films grown on different substrates (glass, PEN, and Cu foil) exhibited similar grain size and crystallinity (Supplementary Fig. 22). However, cracks were observed in the perovskite films deposited on Cu foil, which may be induced by stress release during perovskite crystallization. In addition, the much rougher surface on Cu foil, such as macroscopic cracks formed during the forging process, could be another reason for the worse performance observed in flexible tandems on Cu foil (Supplementary Fig. 23).

## Discussion

In summary, we demonstrated the fabrication of all-perovskite tandems in substrate configuration on both rigid and flexible substrates. With the use of GuaBF_4_ additives, we increased the *V*_*oc*_ and FF of semitransparent WBG PSCs. A PCE of 25.3% was obtained on all-perovskite tandem solar cells in substrate configuration. The substrate-configurated devices exhibited exceptional resistance to oxidization and improved operational stability: the unencapsulated devices remained stable after being exposed to dry air for up to 1000 hours and the encapsulated devices retained 100% of their performance after 600 hours of operation at the MPP under full 1-sun illumination. Furthermore, we fabricated flexible tandem solar cells in substrate configuration with PCEs of 24.1% and 20.3% on PEN/Cu and Cu-foils, respectively. The advantages of substrate configuration in structure and stability provide new possibilities for commercial application of all-perovskite tandems. We found that the low *J*_*sc*_ due to the large reflection is another key factor limiting the performance of substrate-configurated tandems. With proper optical optimization, PCE exceeding 30% will be anticipated^67–69^.

## Methods

### Materials

All materials were used as received without further purification. The organic halide salts (FAI, FABr, MAI, FAI, GuaBF_4_) were purchased from GreatCell Solar Materials (Australia). PEDOT:PSS aqueous solution (Al 4083) was purchased from Heraeus Clevios (Germany). 2PACz (>98.0%) and MeO-2PACz (>98.0%) were purchased from Tokyo Chemical Industry. PbI_2_ (99.99%), PbBr_2_ (99.99%)CsI (99.9%), and CsBr (99.9%) were purchased from TCI Chemicals. SnI_2_ (99.999%) was purchased from Alfa Aesar. SnF_2_ (99%), Indium tin oxide nanocrystals, DMF (99.8% anhydrous), DMSO (99.9% anhydrous), ethyl acetate (99.8% anhydrous), and chlorobenzene (99.8% anhydrous) were purchased from Sigma-Aldrich. C_60_ was purchased from Nano-C (USA). BCP (>99% sublimed) was purchased from Xi’an Polymer Light Technology (China). Ftetrakis(dimethylamino) tin(iv) (99.9999%) was purchased from Ai Mou Yuan Scientific Equipment (Nanjing).

### Perovskite precursor solution

#### Wide-bandgap FA_0.8_Cs_0.2_Pb(I_0.6_Br_0.4_)_3_ perovskite

The precursor solution (1.2 M) of wide-bandgap perovskite (~1.77 eV) was prepared in mixed solvents of DMF and DMSO with a volume ratio of 4:1. The molar ratio of FAI/FABr/CsI/CsBr/PbI_2_/PbBr_2_ was 0.48:0.32:0.12:0.08:0.6:0.4. GuaBF_4_ was added to the precursor solution at optimized concentrations (1.5 mol% of Pb). The precursor solution was stirred at 50 °C for 2 hours and then filtered through 0.22-μm PTFE membrane prior to use.

#### Narrow-bandgap FA_0.7_MA_0.3_Pb_0.5_Sn_0.5_I_3_ perovskite

The precursor solution was prepared in mixed solvents of DMF and DMSO with a volume ratio of 2:1. The molar ratios for FAI/MAI and PbI_2_/SnI_2_ were 0.7:0.3 and 0.5:0.5, respectively. The molar ratio of (FAI + MAI)/(PbI_2_ + SnI_2_) was 1:1. SnF_2_ (10 mol% relative to SnI_2_) was added in the precursor solution. The precursor solution was stirred at room temperature for 2 hours. Tin powders (5 mg mL^−1^) and formamidine sulfinic acid (0.3 mol%) were added to the precursor to reduce Sn^4+^ in the precursor solution and to improve film uniformity. The precursor solution was filtered through a 0.22-μm PTFE membrane before making perovskite films.

### Device fabrication

#### Semitransparent single-junction wide-bandgap solar cells

The pre-patterned indium tin oxide glass substrates were sequentially cleaned using acetone and isopropanol. NiO was deposited by magnetron sputtering on ITO substrates first. Subsequently, the solutions of self-assembled molecule (SAM, 2PACz:MeO-2PACz = 3:1, 1 mmol mL^−1^ in IPA) were spin-coated on the NiO film at 4000 r.p.m. for 20 s, followed by annealing at 100 °C for 1 min in air. After cooling, we transferred the substrates immediately to a nitrogen-filled glovebox for the deposition of perovskite films. The perovskite films were deposited with two-step spin-coating procedures: (1) 2000 r.p.m. for 10 s with an acceleration of 200 r.p.m. s^–1^. (2) 6000 r.p.m. for 40 s with an acceleration of 2000 r.p.m. s^–1^. Chlorobenzene (200 µl) was dropped on the spinning substrate during the second spin-coating step at 20 s before the end of the procedure, followed by annealing at 100 °C for 5 min and 85 °C for 15 min. After cooling down to room temperature, the substrates were transferred to the evaporation system and 12 nm-C_60_ was deposited at the rates of 0.2 Å s^–1^. The substrates were transferred to the ALD system (Veeco Savannah S200) to deposit 15 nm SnO_2_ at a low temperature of 75 °C. The 60 nm-IZO was deposited at room temperature with a background pressure of 4 × 10^−4 ^Pa, working pressure of 0.5 Pa, Argon gas flow of 20 sccm and sputter power of 50 W. A 3-inch IZO target (90wt.% In_2_O_3_ and 10%wt. ZnO) was used for sputtering. Finally, 200 nm Cu was deposited by thermal evaporation as the front grid electrode. Usually, an anti-reflective film was used to reduce light reflection from the surface

#### Substrate configuration all-perovskite tandem solar cells

For rigid devices, the substrate is cleaned glass, while flexible devices are PEN or Cu foil fixed to the glass. 80 nm copper and 10 nm ITO were first deposited on the substrate (glass or PEN) by thermal evaporation and magnetron sputtering, respectively. In particular, for Cu foil tandem solar cells, only 10 nm ITO was needed to be deposited on the Cu surface. PEDOT: PSS was spin-coated on substrates at 4000 rpm for 30 s and annealed on a hotplate at 150 °C for 10 min in ambient air. After cooling, we transferred the substrates immediately to a nitrogen-filled glovebox for the deposition of perovskite films. The perovskite films were deposited with two-step spin-coating procedures: (1) 1000 rpm for 10 s with an acceleration of 200 rpm/s and (2) 4000 rpm for 40 s with a ramp-up of 1000 rpm/s. Ethyl acetate (300 µL) was dropped on the spinning substrate during the second spin-coating step at 20 s before the end of the procedure. The substrates were then transferred on a hotplate and heated at 100 °C for 10 min. After cooling down to room temperature, the substrates were transferred to the evaporation system and 20 nm-C_60_ was deposited at the rates of 0.2 Å s^–1^. In addition to the process described above, we used a scraper to remove the edges of NBG back subcell and then deposited the TRJ and WBG on it. The substrates were transferred to the ALD system to deposit 30 nm SnO_2_ at a low temperature of 75 °C. The 15 nm-ITO was deposited at room temperature with a background pressure of 4 × 10^−4 ^Pa, working pressure of 0.5 Pa, Argon gas flow of 20 sccm, and sputter power of 50 W. A 3-inch ITO target (90wt.% In_2_O_3_ and 10%wt. SnO_2_) was used for sputtering. Subsequent processes are consistent with semitransparent wide-bandgap solar cells.

### Characterization of solar cells

For semitransparent single-junction solar cells, the current density-voltage (*J*–*V*) characteristics were measured using a Keithley 2400 source meter under the illumination of the solar simulator (EnliTech, Class AAA) at the light intensity of 100 mW cm^−2^ as checked with NREL calibrated reference solar cells (KG-5 and KG-0 reference cells were used for the measurements of wide-bandgap and narrow-bandgap solar cells, respectively). Unless otherwise stated, the *J*–*V* curves were all measured in air with a scanning rate of 100 mV s^−1^ (voltage steps of 20 mV and a delay time of 100 ms). The active area was determined by the aperture shade masks (0.0529 cm^2^) placed in IZO side of the solar cells. EQE measurements were performed in ambient air using a QE system (EnliTech) with monochromatic light focused on device pixel and a chopper frequency of 20 Hz. For tandem solar cells, the *J-V* characteristics were carried out under the illumination of a two-lamp high spectral match solar simulator (SAN-EI ELECTRIC, XHS-50S1). EQE measurements were performed in ambient air, and the bias illumination from highly-bright LEDs with emission peaks of 850 and 460 nm were used for the measurements of the front and back subcells, respectively. No bias voltage was applied during the EQE measurements of tandems.

### Stability tests of solar cells

The operational stability tests were carried out under full AM1.5 G illumination (Class AAA, multi-color LED solar simulator, Guangzhou Crysco Equipment Co. Ltd) with an intensity of 100 mW cm^−2^ using a home-build LabVIEW-based MPP tracking system and a *Perturb and observe* method. Unencapsulated samples were tested in a glovebox filled with dry air at a relative humidity of 10–18% and a temperature of 20–25 °C. Encapsulated samples were tested in an ambient air condition with a relative humidity of about 30–50% and a temperature of 20–25 °C. The solar cells were encapsulated with a cover glass and UV epoxy (Three Bond, Japan) which was cured under a UV-LED lamp (peak emission at 365 nm) for 3 min. No UV filter was applied during operation. The dark long-term shelf stability assessments of encapsulated devices were carried out by repeating the *J-V* characterizations over various times and the devices were stored in dry air too.

### First-principles calculations

Electronic structure calculations were carried out within the framework of density functional theory using plane-wave pseudopotential method as implemented in the Vienna Ab initio Simulation Package. The electron-ion interactions were described by using the projected augmented wave pseudopotentials. The *6s*^*2*^*6p*^*2*^ (Pb), *5s*^*2*^*5p*^*5*^ (I), *2s*^*2*^*2p*^*2*^ (C), *2s*^*2*^*2p*^*3*^ (N), *3s*^*2*^*3p*^*3*^ (P), *2s*^*2*^*2p*^*5*^ (F), *2s*^*2*^*2p*^*1*^ (B) were treated explicitly as valence electrons. We used the generalized gradient approximation formulated by Perdew, Burke, and Ernzerhof as exchange correlation functional. To simulate the FA_0.8_Cs_0.2_Pb(I_0.6_Br_0.4_)_3_ perovskite surfaces and crystal boundary, we constructed FAPbI_3_ perovskite 2 × 2 × 4 supercells in the slab structures with a vacuum layer thickness of 15 Å. Structure optimization (including lattice parameters and internal atomic positions) was performed using the conjugate gradient technique until the energies converged to 10^−4^ eV. A kinetic energy cutoff of 400 eV was used for wave-function expansion and only the Γ point was used for Brillouin zone integration. To properly take into account the long-range van der Waals (vdW) interaction that is non-negligible for hybrid perovskites involving organic molecules, the vdW-optB86b functional was adopted.

### Other characterizations

SEM images were obtained using a TESCAN microscope with an accelerating voltage of 2 kV. XRD patterns were collected using a Bruker D8 Advance equipped with a NaI scintillation counter and using monochromatized Copper Kα radiation (λ = 1.5406 Å). XPS analysis was carried out using the Thermo Scientific Al K-Alpha XPS system with energy steps of 0.1 eV. Steady-state PL and time-resolved PL were measured using an Edinburgh FLS980 system. The light was illuminated from the top surface of the perovskite film. For steady-state PL measurements, the excitation source was from a mono-chromated Xe lamp (peak wavelength at 520 nm with a line width of 2 nm). For time-resolved PL, a laser diode (λ = 405 nm) was used for the excitation source with an excitation power density of 0.06 nJ cm^−2^. The PL decay curves were fitted with biexponential function to obtain the fast and slow PL decay lifetimes of *τ*_1_ and *τ*_2_ and the corresponding coefficients of A_1_ and A_2_ of perovskite films, respectively. Then the PL effective decay lifetime *τ*_eff_ was calculated by the following equation: *τ*_eff_ = (A_1_τ_1_ + A_2_τ_2_)/(A_1_ + A_2_). Transient photovoltage decays were measured on a homemade system. A 540 nm green light-emitting diode was used to modulate the *V*_*oc*_ with a constant light bias, and the repetition rate was set to 2000 Hz. The open-circuit voltage transient, induced by the light perturbation, was measured with a digital oscilloscope set to an input impedance of 1 MΩ. The charge recombination lifetime was fitted by a single exponential decay.

### Reporting summary

Further information on research design is available in the Nature Portfolio Reporting Summary linked to this article.

## Supplementary information

Supplementary Information

Description of Additional Supplementary Files

Supplementary Data 1–6

Reporting Summary

## Data Availability

The main data supporting the findings of this study are available within the published article and its Supplementary Information and source data files. Supplementary Figs. 2, 5, 6, 12, 16 are provided in the Supplementary Data 1–6 file. Additional data are available from the corresponding author on request. Source data are provided with this paper.

## Source data

Source Data
