# Supplementary material for: Oxidation-resistant all-perovskite tandem solar cells in substrate configuration
Source: Nat Commun. 2023 Mar 31;14:1819. doi: 10.1038/s41467-023-37492-y (PMC10066323; doi:10.1038/s41467-023-37492-y)
Supplement: Supplementary file 1 — Supplementary Information [file 41467_2023_37492_MOESM1_ESM.pdf]

Supplementary Information

for

Oxidation-resistant all-perovskite tandem solar cells in substrate configuration

Yurui Wang[1†], Renxing Lin[1†], Xiaoyu Wang[2†], Chenshuaiyu Liu[1], Yameen Ahmed[3], Zilong Huang[1], Zhibin Zhang[4], Hongjiang Li[1], Mei Zhang[1], Yuan Gao[1], Haowen Luo[1], Pu Wu[1], Han Gao[1], Xuntian Zheng[1], Manya Li[1], Zhou Liu[1], Wenchi Kong[1], Ludong Li[1], Kaihui Liu[4], Makhsud I. Saidaminov[3] Lijun Zhang[2] & Hairen Tan[1*]

[1]*National Laboratory of Solid State Microstructures, College of Engineering and Applied Sciences, Frontiers Science Center for Critical Earth Material Cycling, Nanjing University, Nanjing 210023, China.*

[2]*State Key Laboratory of Superhard Materials, Key Laboratory of Automobile Materials of MOE, College of Materials Science and Engineering, Jilin University, Changchun, China*

[3]*Department of Chemistry, University of Victoria, Victoria, British Columbia, Canada*

[4]*State Key Laboratory for Mesoscopic Physics, Frontiers Science Center for Nano-optoelectronics, School of Physics, Peking University, Beijing, China*

†These authors contributed equally to this work.

*Corresponding authors. E-mail: hairentan@nju.edu.cn (H.T.)

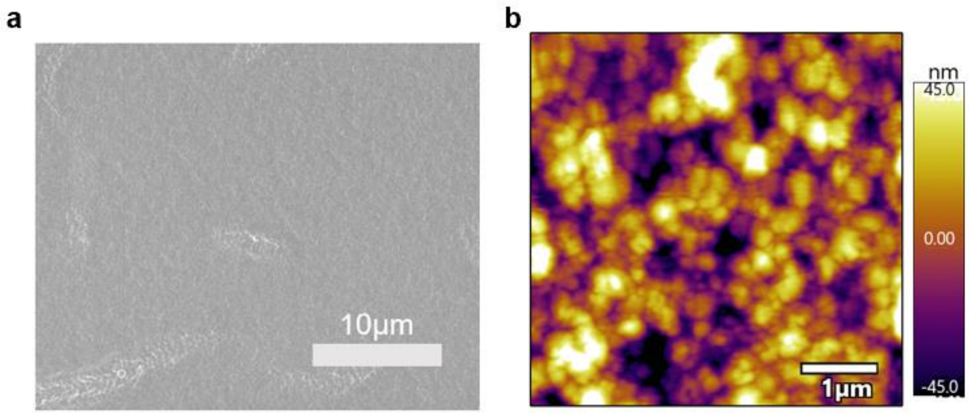

Supplementary Fig. 1 Morphology of WBG perovskite films. a SEM of the surface of ALD-SnO_2 deposited on Glass/ITO/NiO/WBG perovskite/C_{60}. **b** AFM of the surface of ALD-SnO_2 deposited on Glass/ITO/NiO/WBG perovskite/C60. The RMS is 17.2 nm.

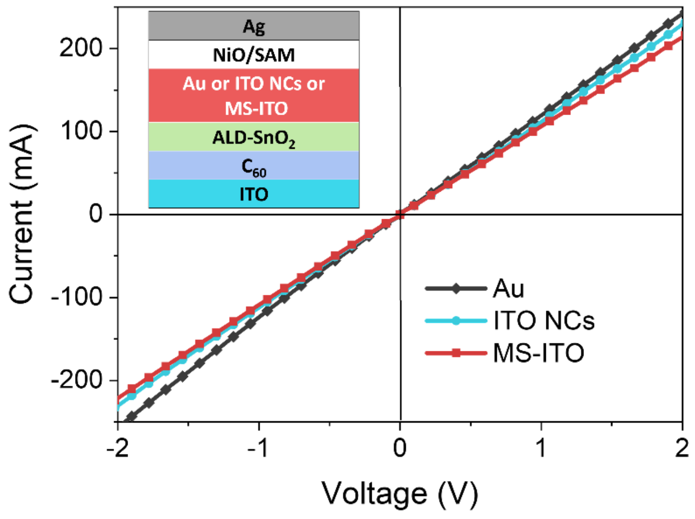

Supplementary Fig. 2 Dark current–voltage curves of different TRJs.

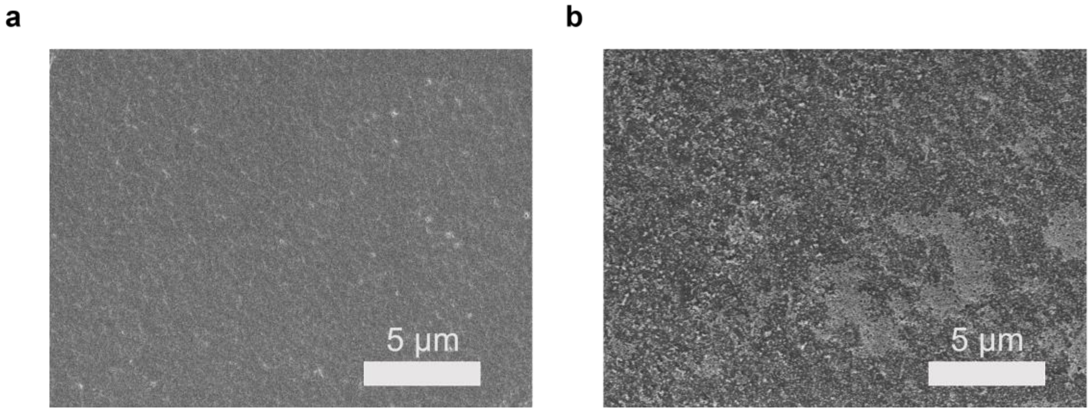

Supplementary Fig. 3 SEM of ITO NCs deposited on different surface. a Glass/ITO/NiO/SAM/WBG/C_{60}/SnO_2 and **b** Glass/ITO/PEDOT: PSS/NBG/C_{60}/SnO_2.

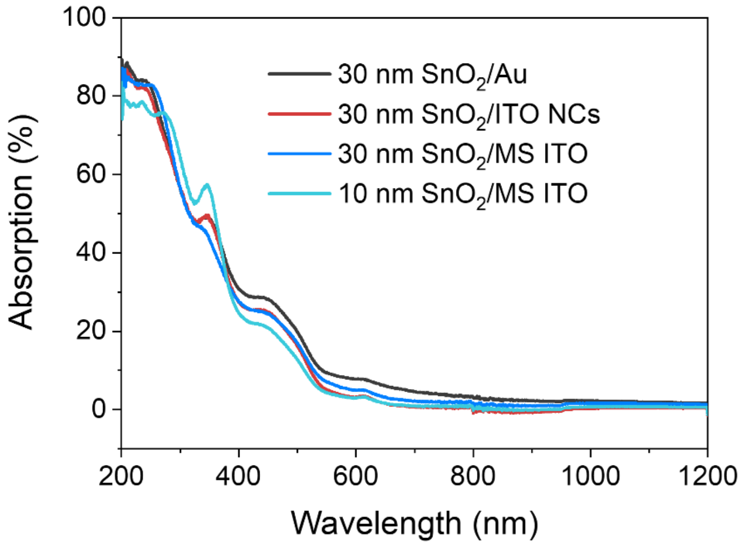

Supplementary Fig. 4 Absorption spectra of different tunneling junction structures.

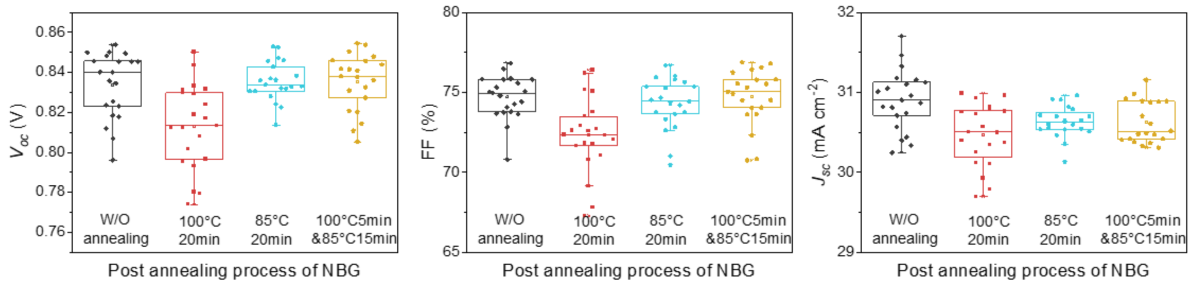

Supplementary Fig. 5 Effect of post annealing conditions on semitransparent NBG PSCs. There are 21 devices for each type, and the box lines indicate the standard deviation, and the centre represents the mean value.

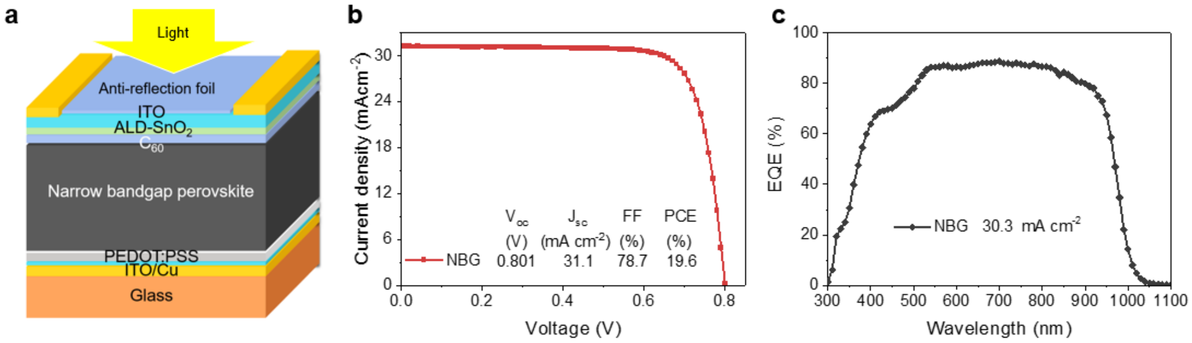

Supplementary Fig. 6 PV performance of NBG perovskite solar cells. a Device structure of NBG perovskite solar cells. **b, c** J–V curves and EQE spectra of NBG perovskite solar cells.

Supplementary Note 1: Mechanism of $GuaBF_4$ in WBG perovskite.

First-principles calculations of WBG perovskite were carried out in the framework of density functional theory (DFT). The electron localization function (ELF) proposed by Becke et al.[1,2] can well describe the degree of electron localization, which we use to determine the bonding behavior. The FA-I (100) crystal surfaces of perovskites were constructed to simulate the surface and grain boundary of WBG perovskites. As shown in **Supplementary Fig. 7**, the electron cloud boundary (green region in **Supplementary Fig. 7**) contact between cations additives and four halogen atoms of perovskite surface reflects the quantitative trend of hydrogen bonding. We found that the Gua^+ can form more hydrogen bonds (4) with perovskite surface halogens than other molecules (FA^+: 3, PA^+: 1, and PEA^+: 0). This is evidenced by the calculated charge density difference (**Supplementary Fig.8**). Multiple hydrogen bonds substantially enhance the stability of halogens on the perovskite surface and inhibit the formation of halogen vacancies[3]. Moreover, we found that the steric effect of Gua^+ on the surface lattice of perovskite is much lower than that of PEA^+ and PA^+. This is evidenced by the greater surface halogen displacement based on balance position (red dashed boxes in **Supplementary Fig. 7**) induced by PEA^+ and PA^+ than Gua^+ and FA^+. This indicates that Gua^+ is more effective in inhibiting halogen vacancy defects caused by halide migration.

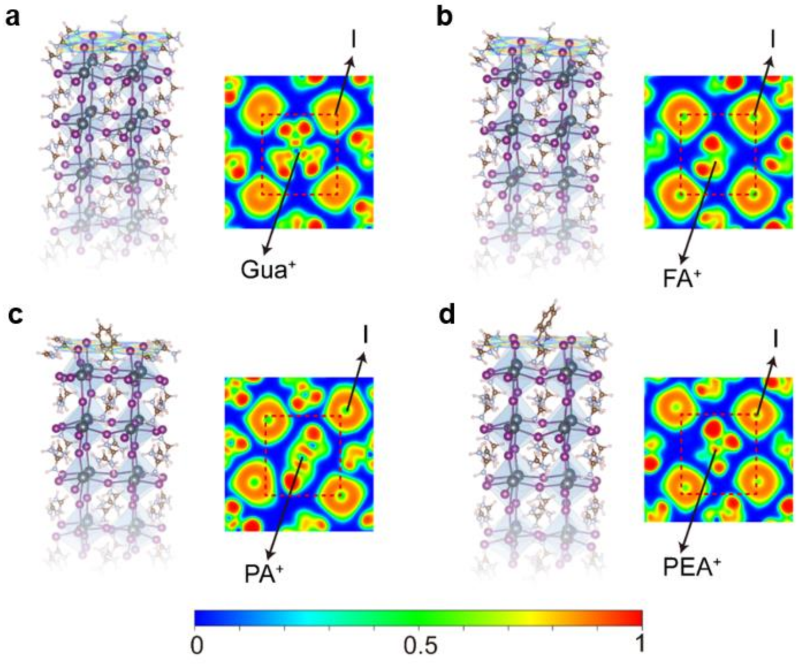

Supplementary Fig. 7: Calculated electron localization function (ELF, two-dimensional data plotting) of perovskite surfaces with different A-site additives. a, Gua+. **b,** FA+ (pure perovskite)**. c,** PA+. **d,** PEA+. The left panel of each subgraph is the optimized structure of perovskites and the plane position for ELF data plotting and the right panel of each subgraph is the two-dimensional ELF data. The values 0 (blue) and 1 (red) represent perfect delocalization and localization for electrons, respectively. The green part (value ~0.5) approximately represents the boundary of the electron cloud. The red dashed boxes mark the halogen equilibrium position in the pure perovskite.

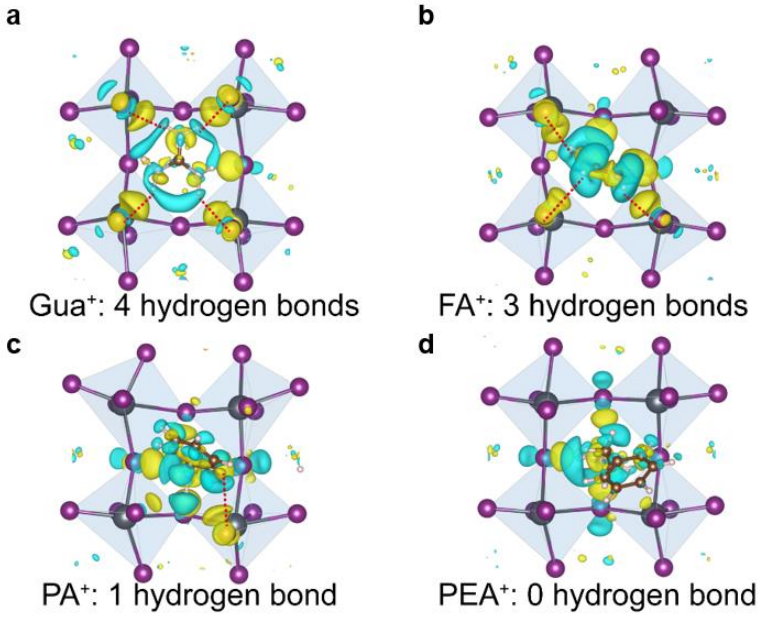

Supplementary Fig. 8: Calculated charge density difference (three-dimensional data plotting), showing the chemical interactions between the perovskite surface and the A-site additives. a, Gua+. **b,** FA+ (pure perovskite)**. c,** PA+. **d,** PEA+. The hydrogen bonds can be noted by the red dashed lines. Blue and yellow show regions of space that are depopulated or populated by electrons, respectively (as a result of the surface-additive bonding).

To investigate the effect of anion additives on the bonding behavior of Pb^{2+}, we plotted one-dimensional ELF data for six different coordination directions of the surface octahedral of perovskite (**Supplementary Fig. 9**). For pure perovskite (**Supplementary Fig. 9**), the Pb-I

bond is dominated by ionic bonding components. The pseudo-halogen (BF_4^-, PF_6^-) introduces a new Pb-F bond into perovskite. The Pb-F bonds cause a redistribution of the ELF value (red lines in **Supplementary Fig. 9b** and **Supplementary Fig. 9c**). The increase in the percentage of ELF < 0.5 part indicates the enhancement of the covalent bonding components. We also found that the Pb-F bond introduced by BF_4^- (2.89 Å) is shorter than that introduced by PF_6^- (2.98 Å). This indicates that the Pb-F bond introduced by BF_4^- is stronger. Therefore, pseudo-halogen is good passivator for halogen vacancies, and the BF_4^- has better passivation ability than PF_6^-.

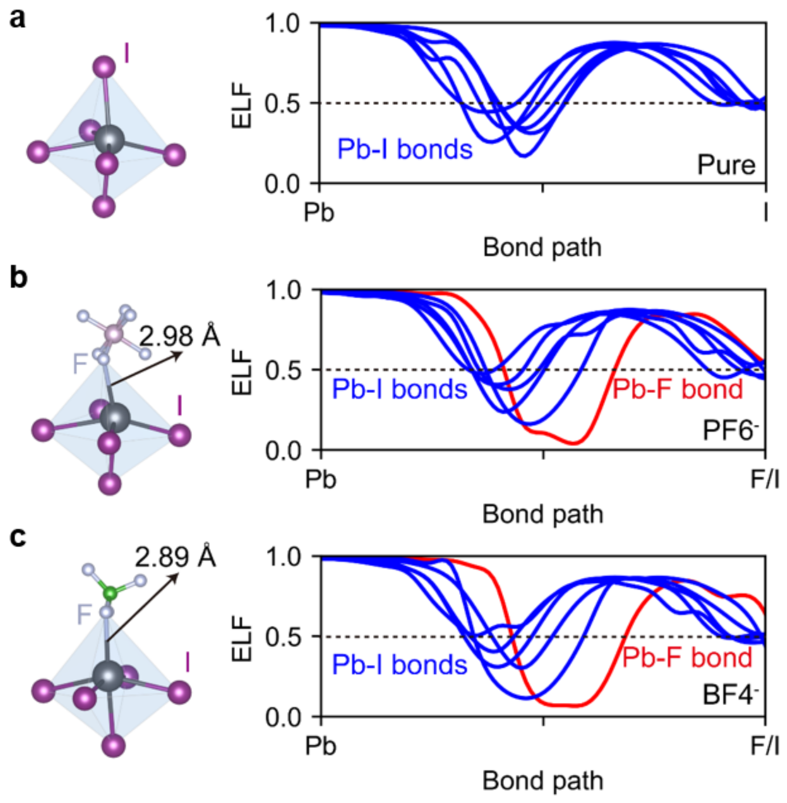

Supplementary Fig. 9: Calculated electron localization function (ELF, one-dimensional data plotting) along the direction of the octahedral bond length (Pb-F/I bonds) on the perovskite surface with different X-site additives. a, I- (pure perovskite). **b,** PF_6^-. **c,** BF_4^-. The left panel of each subgraph is the optimized structure of the surface octahedral of perovskite that contains X-site additives. The right panel of each subgraph is one-dimensional ELF data along the six bonding directions in the octahedral. The endpoints of the horizontal axis represent the centers of the two atoms that form the chemical bonds (Pb-F/I bonds). Blue and red show

the ELF values along the Pb-I and Pb-F bonding directions, respectively. The ELF > 0.5 part represents the ionic bond contribution and the ELF < 0.5 part represents the covalent bond contribution.

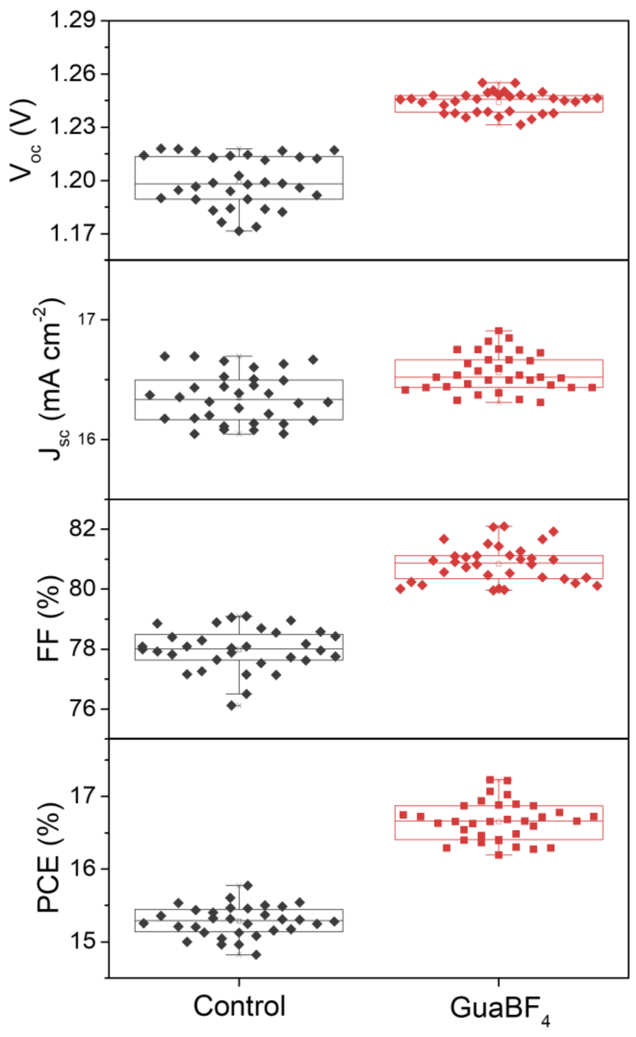

Supplementary Fig. 10 Performance distribution of control and $GuaBF_4$ semitransparent WBG perovskite solar cells. There are 40 devices for each type, and the box lines indicate the standard deviation, and the centre represents the mean value.

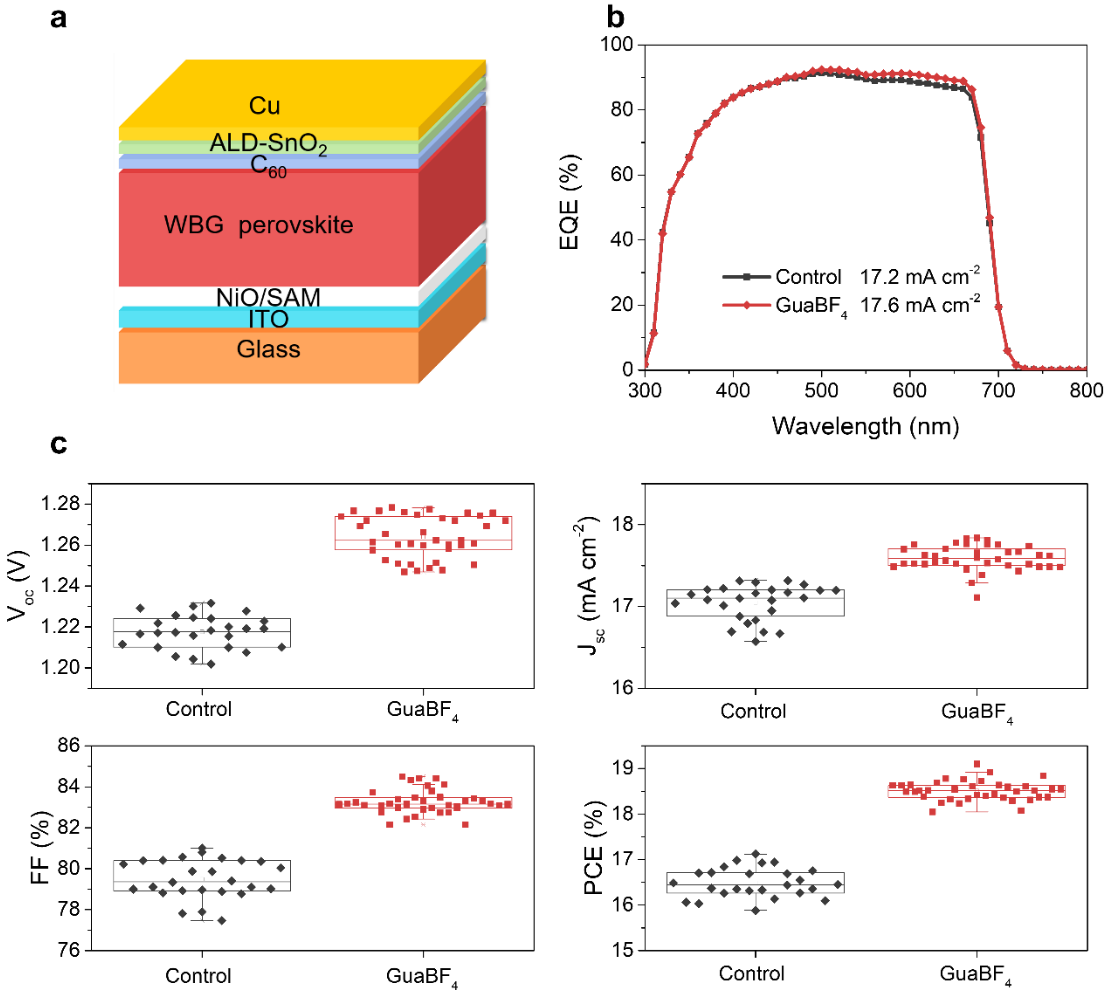

Supplementary Fig. 11 PV performance of WBG perovskite solar cells. a Schematic diagram of WBG perovskite solar cells. **b, c** EQE spectra and performance distribution of control and $GuaBF_4$ WBG perovskite solar cells.

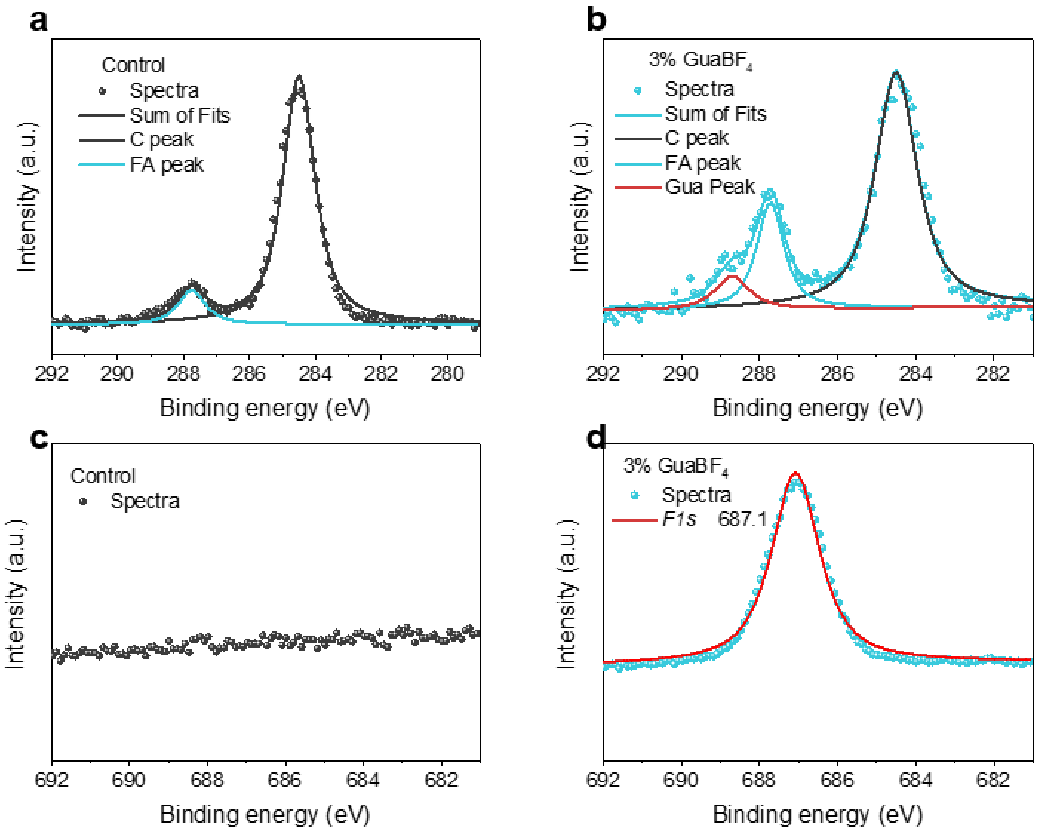

Supplementary Fig. 12 XPS spectra of control and $GuaBF_4$ perovskite films. a, b *C1s* in control and $GuaBF_4$ perovskite films. **c, d** *F1s* in control and $GuaBF_4$ perovskite films.

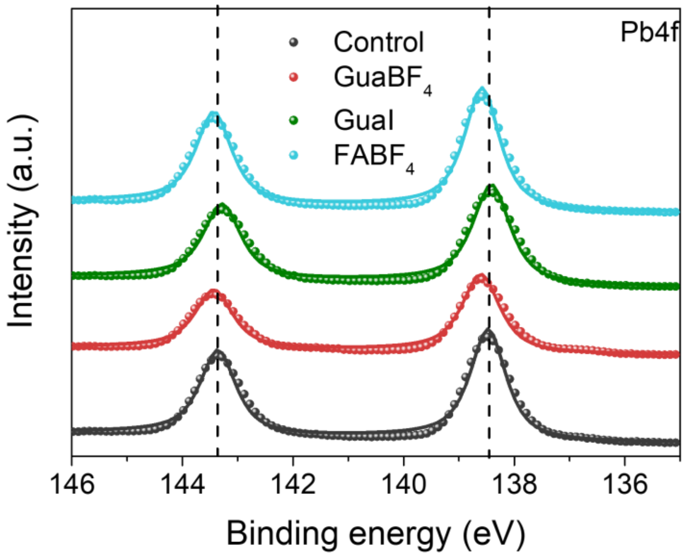

Supplementary Fig. 13 The Pb4f$_{5/2}$ and Pb4f$_{7/2}$ XPS spectra of perovskite films with different additives.

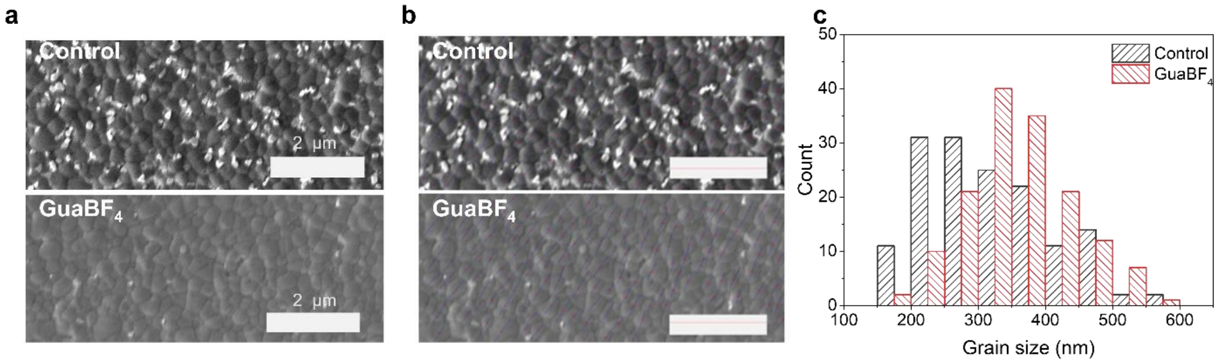

Supplementary Fig. 14 SEM image of control and $GuaBF_4$ perovskite films. a, b SEM. **c** Statistics of control and $GuaBF_4$ perovskite grain size. The average grain size is 319 nm for control and 356 nm for $GuaBF_4$.

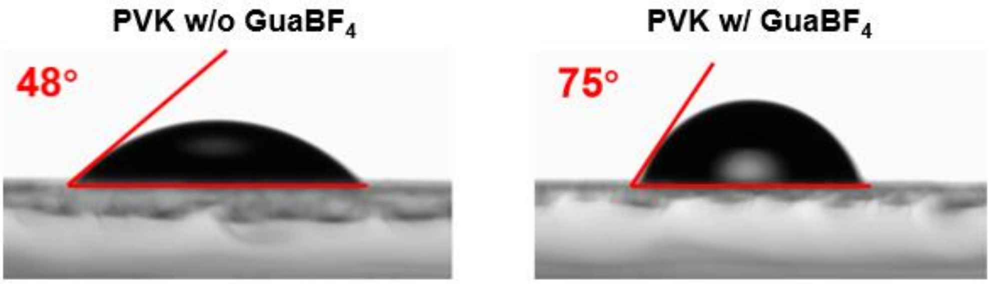

Supplementary Fig. 15 Contact angle of control and $GuaBF_4$ perovskite films.

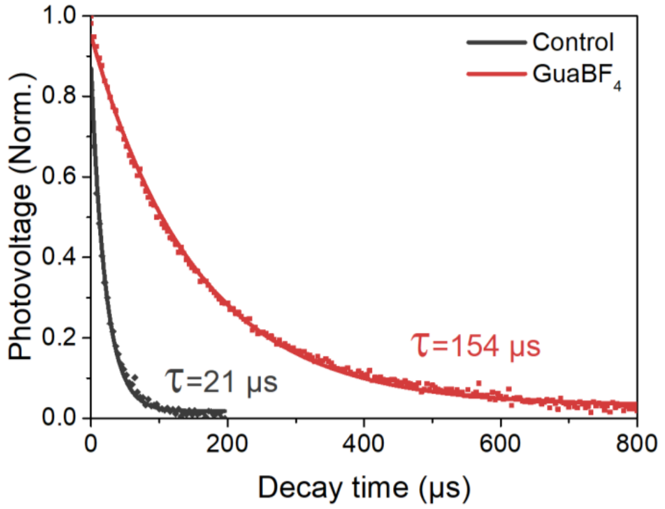

Supplementary Fig. 16 Transient photovoltage decay of control and $GuaBF_4$ WBG perovskite solar cells. The recombination lifetime (τ) is monoexponentially fitted.

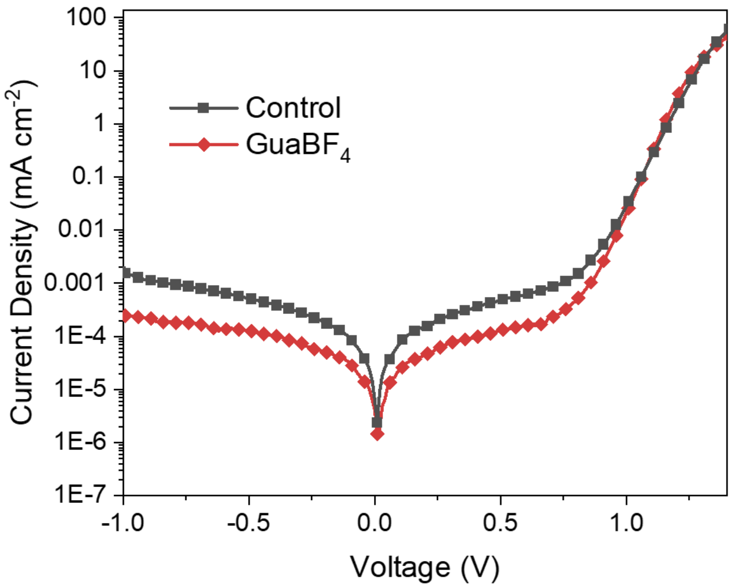

Supplementary Fig. 17 Dark J-V cures of device with and without GuaBF$_4$.

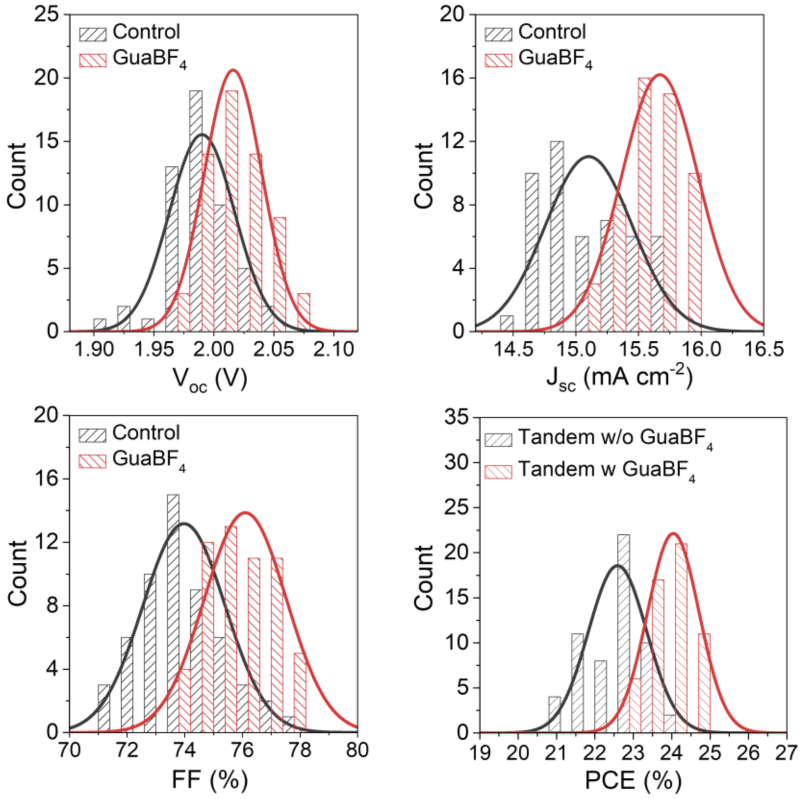

Supplementary Fig. 18 PV performance distribution of control and $GuaBF_4$ substrate-configured all-perovskite tandem solar cells.

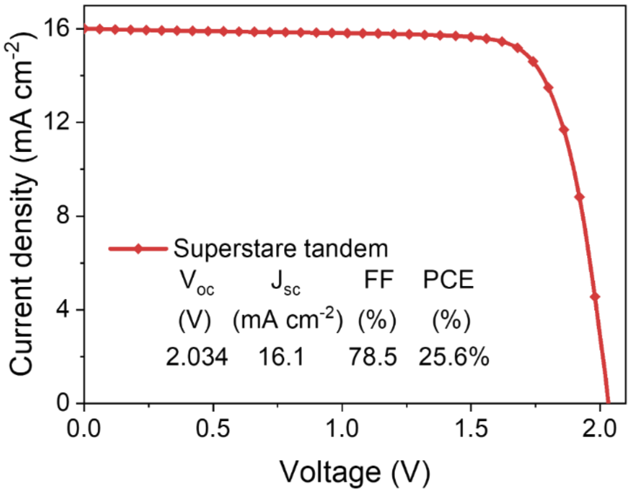

Supplementary Fig. 19 The J–V curve of superstrate-configured all-perovskite tandem solar cell.

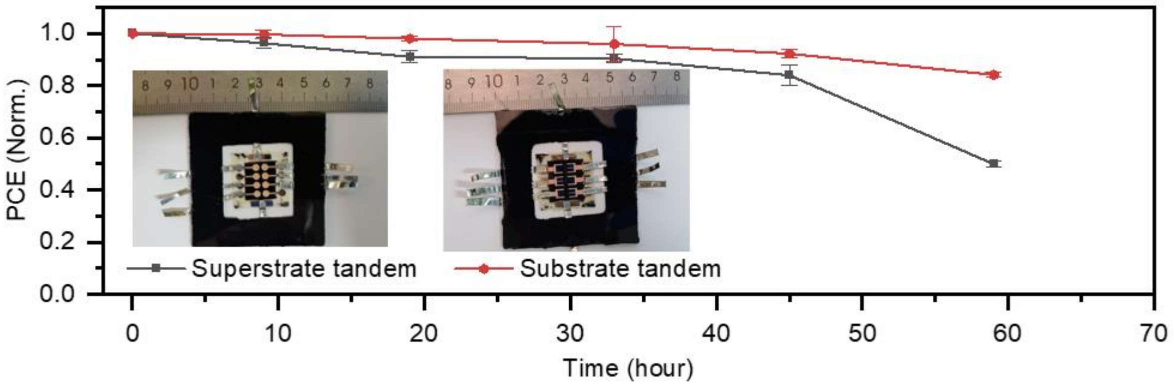

Supplementary Fig. 20 Stability of tandem devices at 85 °C and relative humidity of 85%.

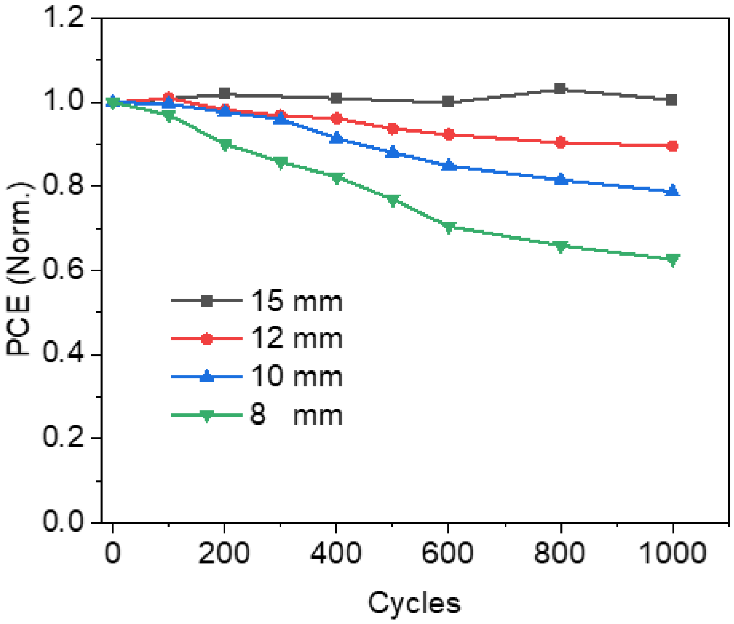

Supplementary Fig. 21 Bending tests of flexible substrate-configurated tandems under different bending radius.

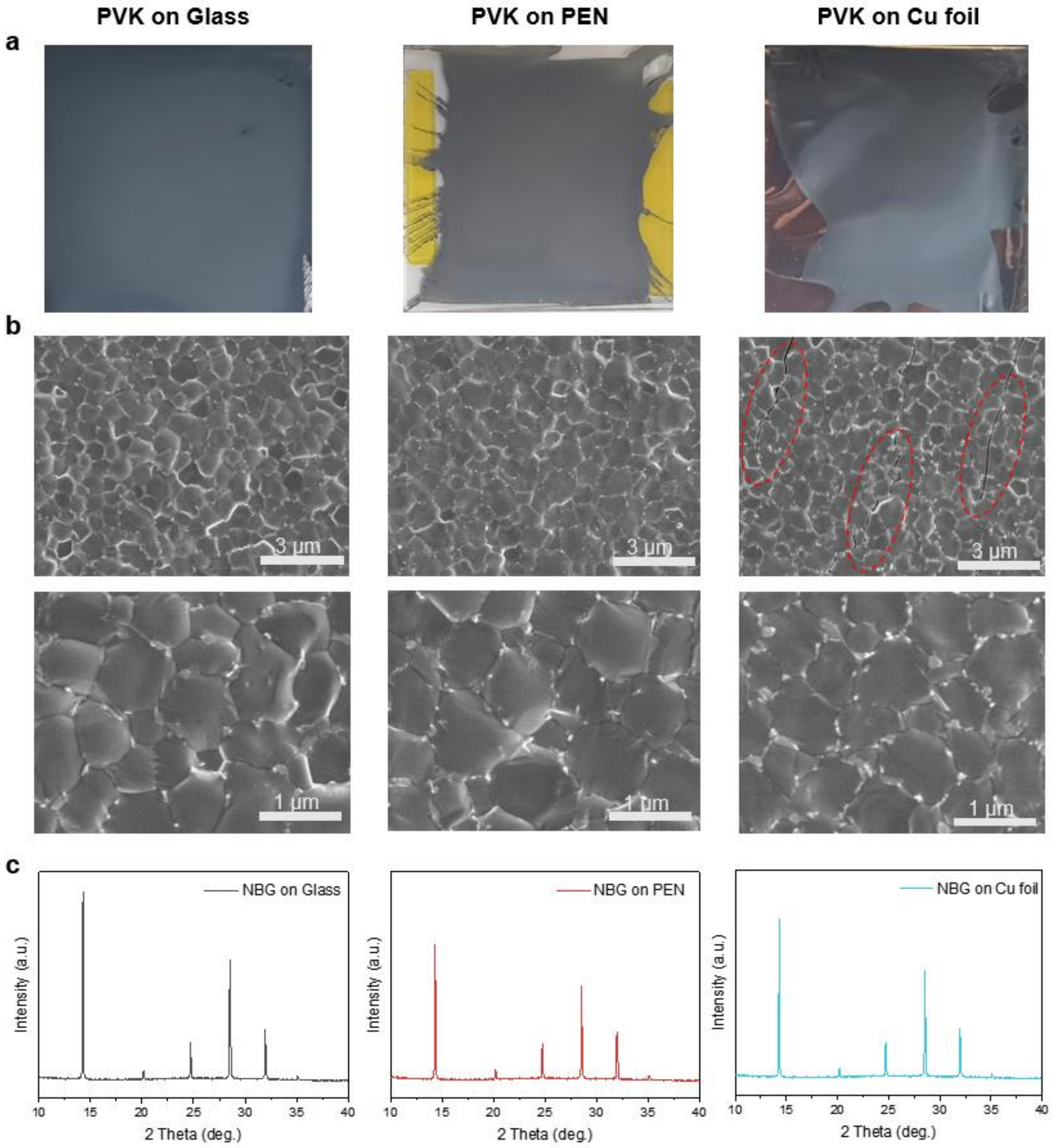

Supplementary Fig. 22 Characteristics of NBG perovskite deposited on different substrates. a, Photographs**, b,** SEM and **c,** XRD of films deposited on glass, PEN and Cu foils.

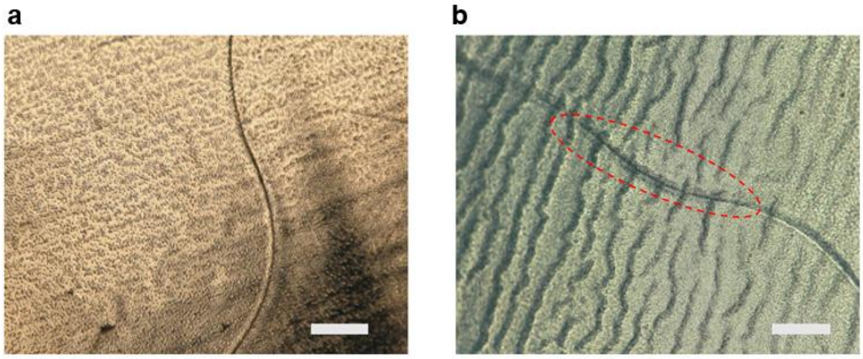

Supplementary Fig. 23 Optical microscope images of bare Cu foil and perovskite films deposited on Cu-foil. a, bare Cu-foil and **b,** NBG perovskite film on Cu foil/ITO/PEDOT:PSS. Perovskite is deposited along cracks in the substrate in the dotted frame area.

Supplementary Note 2: Dark stability of tandem devices stored in high humidity environment.

We tested the high humidity stability of unencapsulated tandem devices. We fabricated three sets of devices: superstrate-configured tandems with $GuaBF_4$ in WBG, $GuaBF_4$ free substrate-configured tandem, and substrate-configured tandems with $GuaBF_4$ in WBG.

In **Supplementary Fig. 24**, we show pictures of the tandem device before and after stored in high humidity environment (RH=85%). After 25 h high humidity treatment, the morphology of superstrate-configured tandem changes obviously, mainly due to the degradation of both copper electrode and perovskite layer. For substrate-configured tandems, damage was not obviously observed even after 102 h. This is mainly due to the additional water barrier effect of the extra IZO and the better water resistance of the FACs WBG perovskite compared to the MA-contained NBG perovskite[4–6].

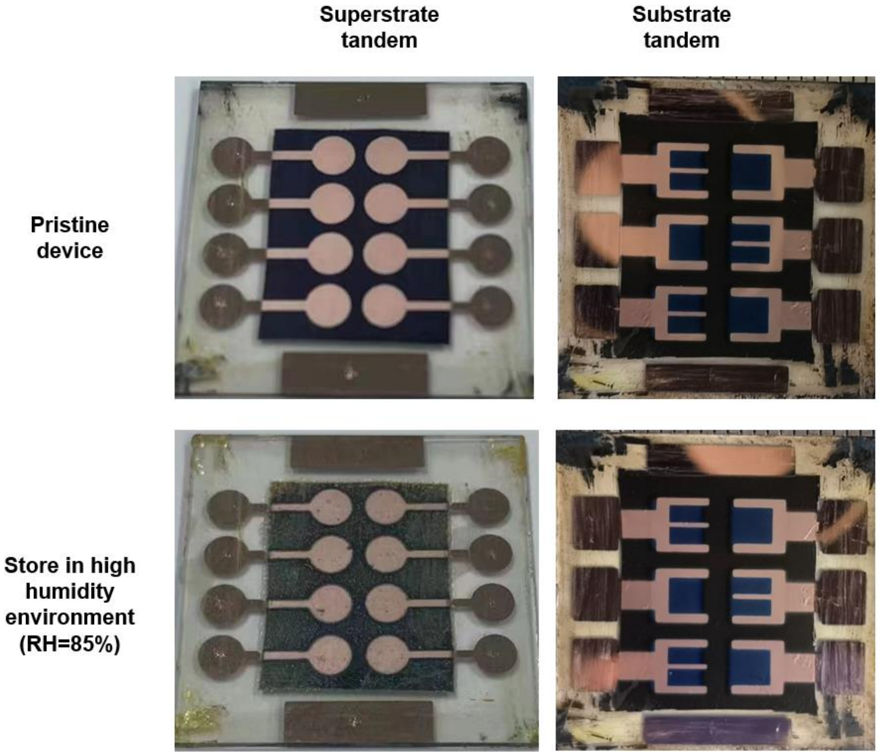

Supplementary Fig. 24 Pictures of the tandem devices before and after stored in high humidity environment. The storage time is 25 hours for superstrate-configured tandems and

102 hours for substrate-configured tandems.

Supplementary Fig. 25 indicates that the improved humidity resistance is ascribed to the advantages of device structure in substrate-configured tandems. During the first 25 hours, the performance of the tandems with a substrate configuration retained more than 90% during the first 25 hours, whereas the tandems with a superstructure configuration degraded significantly below 70%. In addition, we noted that substrate-configure tandem with additives in WBG had slightly better stability over longer monitoring times, owing to the better hydrophobicity of $GuaBF_4$ films observed in **Supplementary Fig. 15**.

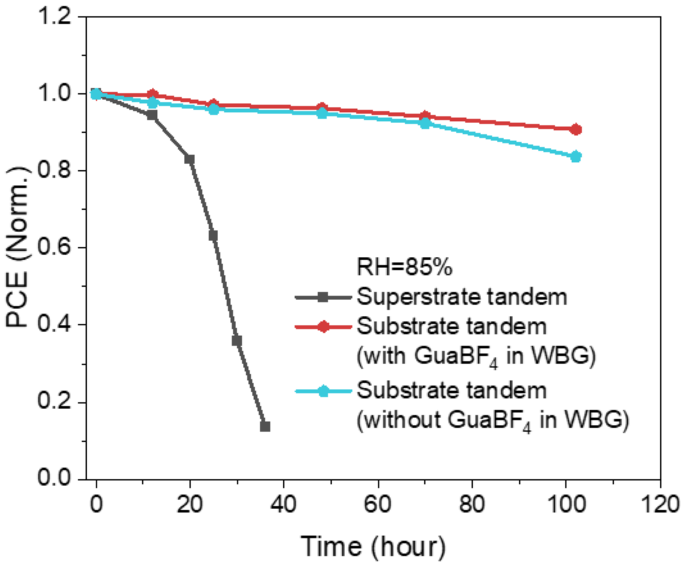

Supplementary Fig. 25 Dark stability of tandem devices stored in high humidity environment. Unencapsulated devices. Place in an airtight glove box with a relative humidity of 85%.

In superstrate-configured tandem, all PV parameters have huge declines accompanied by larger JV hysteresis after 25h humidity treatment (**Supplementary Fig. 26**, hysteresis is defined as ($PCE_{Reverse}$ – $PCE_{Forward}$)/$PCE_{Reverse}$). This is because moisture (and oxygen in air) has been observed to damage the lateral NBG perovskite, possibly seriously affecting charge transport at the interface. In the superstrate-configured tandem, we observed a slightly decreased performance even after 102 h humidity treatment, which could be mainly caused by moisture-

induced halogen phase separation in WBG perovskite[5].

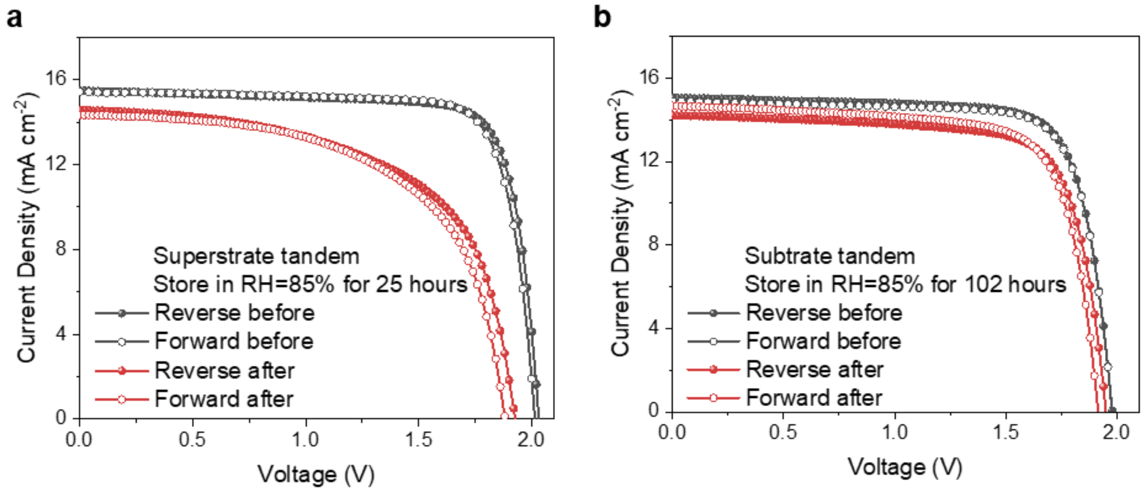

Supplementary Fig. 26 J-V cures of tandem devices before and after high humidity treatment. a, Superstrate-configured tandem. **b,** Substrate-configured tandem."

Supplementary Note 3: Light soaking stability of tandem devices.

The device was encapsulated with butyl rubber and glass, and the light soaking stability was monitored under a LED light with the illumination of 1-sun. **Supplementary Fig. 27** shows pictures of the device before and after light soaking for 120 hours. No significant morphological changes were observed in both the superstrate- and the substrate-configured tandems.

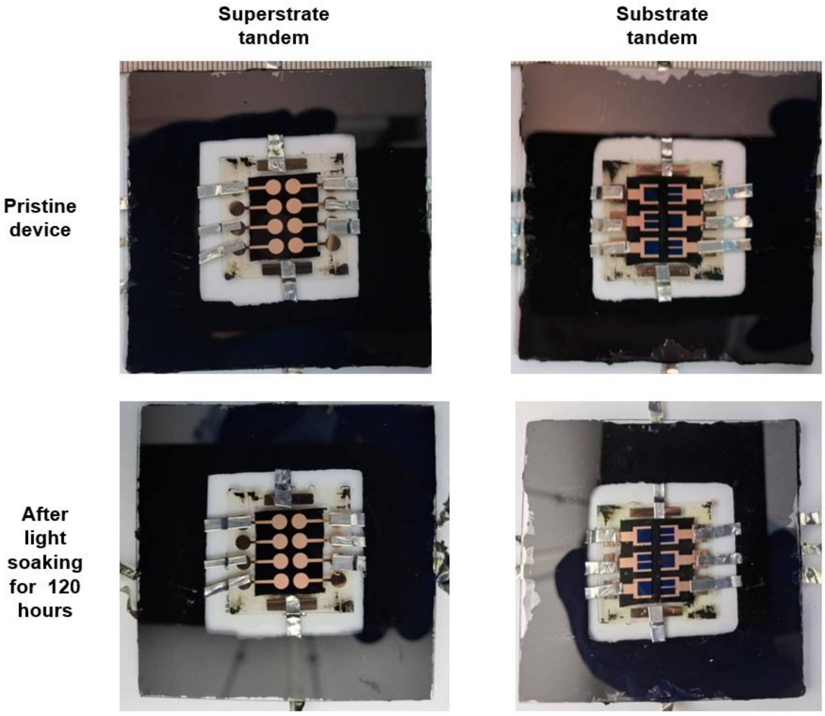

Supplementary Fig. 27 Pictures of the tandem devices before and after light soaking.

Overall, there was a similar decline in PCE and FF for both superstrate- and substrate-configured tandem devices (**Supplementary Fig. 28**). And no apparent change in hysteresis was observed. The small increases in V_{oc} can be explained by that light soaking can reduce the density of charged defects through charge trappinng[7,8].

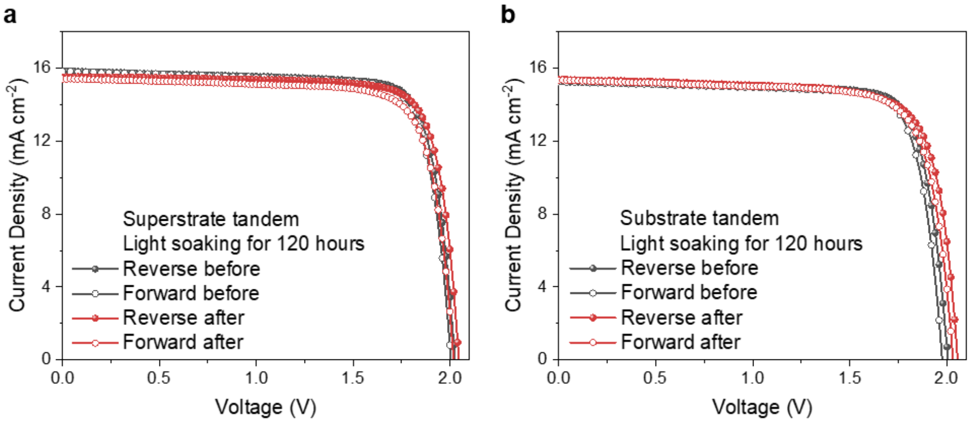

Supplementary Fig. 28 Light soaking stability of tandem devices. a, *J-V* cures of superstrate-configured tandem devices before and after light soaking. **b,** *J-V* cures of substrate-configured tandem devices before and after light soaking. Encapsulated devices. Place under LED lights with 1-sun illumination."

Supplementary References

1. Becke, A. D. & Edgecombe, K. E. A simple measure of electron localization in atomic and molecular systems. *J. Chem. Phys.* **92**, 5397–5403 (1990).
2. Silvi, B. & Savin, A. Classification of chemical bonds based on topological analysis of electron localization functions. *Nature* **371**, 683–686 (1994).
3. Kim, Y.-H. *et al.* Comprehensive defect suppression in perovskite nanocrystals for high-efficiency light-emitting diodes. *Nat. Photonics* **15**, 148–155 (2021).
4. Lee, C. *et al.* Influence of ZnO buffer layer thickness on the electrical and optical properties of indium zinc oxide thin films deposited on PET substrates. *Ceram. Int.* **34**, 1093–1096 (2008).
5. Eperon, G. E. *et al.* Perovskite-perovskite tandem photovoltaics with optimized band gaps. *Science.* **354**, 861–865 (2016).
6. Li, C. *et al.* Low-bandgap mixed tin–lead iodide perovskites with reduced methylammonium for simultaneous enhancement of solar cell efficiency and stability. *Nat. Energy* **5**, 768–776 (2020).
7. Zhao, C. *et al.* Revealing Underlying Processes Involved in Light Soaking Effects and Hysteresis Phenomena in Perovskite Solar Cells. *Adv. Energy Mater.* **5**, 1500279 (2015).
8. Wu, X. *et al.* Control over Light Soaking Effect in All-Inorganic Perovskite Solar Cells. *Adv. Funct. Mater.* **31**, 2101287 (2021).
