# Supplementary material for: Oxidation-resistant all-perovskite tandem solar cells in substrate configuration
Source: Nat Commun. 2023 Mar 31;14:1819. doi: 10.1038/s41467-023-37492-y (PMC10066323; doi:10.1038/s41467-023-37492-y)
Supplement: Supplementary file 2 — Description of Additional Supplementary Files [file 41467_2023_37492_MOESM2_ESM.pdf]

Description of Additional Supplementary Files

Supplementary Data 1-6

Supplementary Data 1
The data of dark current–voltage curves shown in Supplementary Fig. 2.

Supplementary Data 2
PV parameters of NBG PSCs shown in Supplementary Fig. 5.

Supplementary Data 3
The J-V curve and EQE of NBG PSCs shown in Figure 6.

Supplementary Data 4
PV parameters of WBG PSCs shown in Supplementary Fig. 10.

Supplementary Data 5
XPS data of control and GB films shown in Supplementary Fig. 12.

Supplementary Data 6
TPV data of control and GB devices shown in Supplementary Fig. 16.

Source Data

PV parameters of WBG and NBG PSCs shown in Figure. 1c and Figure 1d. The J-V curves of the tandem device shown in Figure 1e.

The J-V curve, XRD, XPS, PL, TRPL, and ideal factor data shown in Figure 2.

The J-V curve, MPP, and EQE of the tandem device shown in Figure 3.

Dark-state stability data, EQE and MPP data shown in Figure 4.

The J-V curve and EQE of the flexible tandem device shown in Figure 5.
