# Supplementary material for: Oxidation-resistant all-perovskite tandem solar cells in substrate configuration
Source: Nat Commun. 2023 Mar 31;14:1819. doi: 10.1038/s41467-023-37492-y (PMC10066323; doi:10.1038/s41467-023-37492-y)
Supplement: Supplementary file 4 — Reporting Summary [file 41467_2023_37492_MOESM4_ESM.pdf]

Corresponding author(s): Hairen Tan

Solar Cells Reporting Summary

Nature Research wishes to improve the reproducibility of the work that we publish. This form is intended for publication with all accepted papers reporting the characterization of photovoltaic devices and provides structure for consistency and transparency in reporting. Some list items might not apply to an individual manuscript, but all fields must be completed for clarity.

For further information on Nature Research policies, including our data availability policy, see Authors & Referees.

Experimental design

Please check: are the following details reported in the manuscript?

1. Dimensions

Item	Yes/No	Response
Area of the tested solar cells	☒ Yes ☐ No	Aperture areas of 0.0529 and 0.049 cm2 were used in this work.
Method used to determine the device area	☒ Yes ☐ No	Black metal aperture masks were used during the J-V measurements.

2. Current-voltage characterization

Item	Yes/No	Response
Current density-voltage (J-V) plots in both forward and backward direction	☒ Yes ☐ No	Provided in this manuscript.
Voltage scan conditions *For instance: scan direction, speed, dwell times*	☒ Yes ☐ No	Provided in Method section.
Test environment *For instance: characterization temperature, in air or in glove box*	☒ Yes ☐ No	Provided in Method section.
Protocol for preconditioning of the device before its characterization	☐ Yes ☒ No	No preconditioning was used in this work.
Stability of the J-V characteristic *Verified with time evolution of the maximum power point or with the photocurrent at maximum power point; see ref. 7 for details.*	☒ Yes ☐ No	Stabilized PCE of tandem solar cells were provided.

3. Hysteresis or any other unusual behaviour

Item	Yes/No	Response
Description of the unusual behaviour observed during the characterization	☒ Yes ☐ No	Very minor hysteresis was observed for devices herein.
Related experimental data	☒ Yes ☐ No	J-V curves under reverse and forward scans were provided.

4. Efficiency

Item	Yes/No	Response
External quantum efficiency (EQE) or incident photons to current efficiency (IPCE)	☒ Yes ☐ No	EQE curves were provided.
A comparison between the integrated response under the standard reference spectrum and the response measure under the simulator	☒ Yes ☐ No	The integrated Jsc values from QE were consistent with Jsc values from J-V measurements.
For tandem solar cells, the bias illumination and bias voltage used for each subcell	☒ Yes ☐ No	Stated in Method section.

5. Calibration

Item	Yes/No	Response
Light source and reference cell or sensor used for the characterization	☒ Yes ☐ No	Stated in Method section.
Confirmation that the reference cell was calibrated and certified	☒ Yes ☐ No	The reference cells were calibrated by NREL and explained in Method.

Calculation of spectral mismatch between the reference cell and the devices under test

☐ Yes ☒ No

The light spectrum used for measurements matches well with the reference silicon cell, and we did not calculate the spectral mismatch factor between the reference cell and the tested devices

6. Mask/aperture

Size of the mask/aperture used during testing

☒ Yes ☐ No

Metal aperture masks with areas of 0.0529 cm2 and 0.049 cm2 were used for testing.

Variation of the measured short-circuit current density with the mask/aperture area

☐ Yes ☒ No

We measured all devices with masks.

7. Performance certification

Identity of the independent certification laboratory that confirmed the photovoltaic performance

☐ Yes ☒ No

We do not certify the device

A copy of any certificate(s)
Provide in Supplementary Information

☐ Yes ☒ No

We do not certify the device

8. Statistics

Number of solar cells tested

☒ Yes ☐ No

Stated in the manuscript.

Statistical analysis of the device performance

☒ Yes ☐ No

Stated in the manuscript.

9. Long-term stability analysis

Type of analysis, bias conditions and environmental conditions
For instance: illumination type, temperature, atmosphere humidity, encapsulation method, preconditioning temperature

☒ Yes ☐ No

Stated in the manuscript.
